# Natural Products Research in China From 2015 to 2016

**DOI:** 10.3389/fchem.2018.00045

**Published:** 2018-03-20

**Authors:** Haishan Liu, Guoliang Zhu, Yaqin Fan, Yuqi Du, Mengmeng Lan, Yibo Xu, Weiming Zhu

**Affiliations:** ^1^School of Medicine and Pharmacy, Ocean University of China, Qingdao, China; ^2^Laboratory for Marine Drugs and Bioproducts of Qingdao National Laboratory for Marine Science and Technology, Qingdao, China

**Keywords:** natural products, China, structure, bioactivity, novel skeleton

## Abstract

This review covers the literature published by chemists from China during the 2015–2016 on natural products (NPs), with 1,985 citations referring to 6,944 new compounds isolated from marine or terrestrial microorganisms, plants, and animals. The emphasis is on 730 new compounds with a novel skeleton or/and significant bioactivity, together with their source organism and country of origin.

## Introduction

Natural products (NPs) play an indispensable role in the drug development process and have provided various molecules for research over the years. Among the 1,211 small-molecule drugs approved from 1981 to 2014, 33% were based on NPs or their derivatives (Newman and Cragg, 2016).

Using natural resources to treat disease was the wisdom of the ancient Chinese and there is a long history of Chinese people using traditional Chinese medicines (TCMs) to treat a variety of diseases. NPs research in China originated in the 1920s and began with separation and identification of the main components of TCMs, such as *Panax notoginsen* (Zhao, 1937), *Brucea javanica* (Xu and Pan, 1955) as well as *Aconitum carmichaeli* (Zhao, 1936). The improvement of science and technology after the founding of the People's Republic of China in 1949, along with the establishment of new methods (Colegate and Molyneux, 2007) involved in detection and analysis of compounds led to the rapid development and great achievements of China's NPs research. In particular, huperzine A (Wang et al., 2006), a novel acetylcholinesterase (AChE) inhibitor derived from the Chinese medicinal herb *Huperzia serrata*, has been used to treat Alzheimer's disease and Youyou Tu was awarded the Nobel Prize in Physiology or Medicine in 2015 for her major contribution to the discovery of artemisinin (Tu, 2011).

By the 1980s, chemists from China not only focused on NPs isolated from TCMs or other terrestrial sources, but also expanded the research to marine natural products (MNPs). The number of new MNPs discovered in China increased exponentially since the 1990s, making China the second most country involved in MNPs discovery behind Japan (Blunt et al., 2016). Taken together, these breakthroughs led to the renewal of NPs research in China.

Reviews of NPs are generally based on their producers [mangrove (Wu et al., 2008), *Paeonia* (Zhao et al., 2016), fungi (Wang et al., 2011), etc.], chemical structures [sesquiterpenoids (Wang et al., 2014), triterpenoids (Xiao et al., 2008), lignans (Zhang et al., 2014), alkaloids (Ma et al., 2016e), etc.] and bioactivities [analgesic activity (Xiao et al., 2016a), antiviral activity (Jiang et al., 2010), cytotoxicity (Wang et al., 2014), etc.] while few reviews are based on the country where the authors come from. According to our current research, more than 6,500 papers have been published by chemists from China in the past 2 years covering all aspects related to NPs, and of these, 1,985 were related to new NPs, a total of about 30%. This review covers the literature published from 2015 to 2016 with 1,985 citations (1,103 for 2015 and 882 for 2016) referring to new NPs isolated from terrestrial- or marine-sourced animals, plants, and microorganisms. In total, 6,944 new small-molecule compounds are summarized (3,891 for 2015 and 3,053 for 2016). The emphasis is on new compounds with a novel skeleton or/and significant biological activity. Pharmaceutical data are directly cited from the original paper, and only comparable or more potent activity relative to the positive control is defined as significant activity. For the cytotoxicity values, significant activity means a half maximal inhibitory concentration (IC_50_) value below 1 μM or 0.5 μg/mL. Chemical structures, together with classifications, taxonomic origins, locations of collections, and biological activities are described in detail. The numbers for all structures in this review are shown in non-italicized bold font.

## Marine microorganisms

A total of 612 novel NPs was isolated from marine microorganisms in the last 2 years. The percentage of compounds with new skeletons from marine bacteria (19.5%) in this review is much higher than average (5.1%), the percentage of bioactive compounds from marine fungi (36.3%) is also higher than the average (28.4%). A total of 46 references related to 101 NPs with a novel skeleton or/and significant bioactivity are listed below.

### Marine-derived bacteria (including mangrove-derived bacteria)

#### Marine-derived bacteria (except mangrove-derived bacteria)

**Figure 1:**
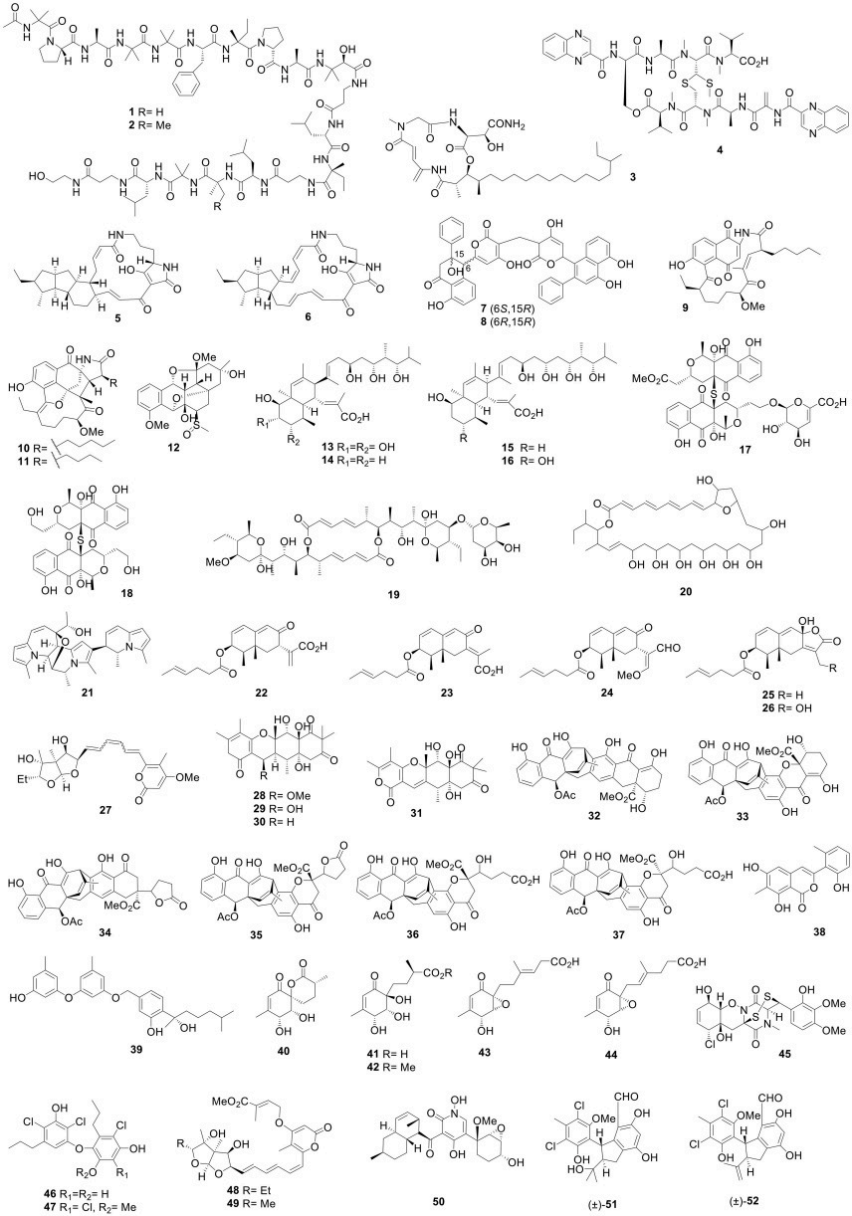
Structures of compounds **1-52**.

Microbacterins A (**1**) and B (**2**) (Figure 1), two new peptaibols, were produced by the deep-sea actinomycete *Microbacterium sediminis* sp. nov. YLB-01(T). Microbacterin B (**2**) displayed potent inhibitory activity against BGC-823 cells with an IC_50_ value of 1.03 μM (Liu et al., 2015b). The marine sediment-derived *Micromonospora* sp. FIM02-523 (Fujian, China) produced a new depsipeptide, rakicidin B1 (**3**), which possessed inhibitory activity against HCT-8, MGC803, A549, and A375 cells with IC_50_ range of 0.11–0.64 μM (Lin et al., 2016b). The marine sponge-derived *Streptomyces* sp. LS298 (*Gelliodes carnosa*, Lingshui Bay, Hainan, China) produced a new analog of echinomycin, quinomycin G (**4**), which exhibited remarkable cytotoxic activity against the Jurkat cells with the IC_50_ value of 0.414 μM (Zhen et al., 2015). Six new polycyclic tetramate macrolactams (PTMs), pactamides A–F, were isolated from the marine-derived *Streptomyces pactum* SCSIO 02999 by activating a PTM gene cluster, among which pactamides A (**5**) and C (**6**) displayed potent cytotoxicities against SF-268, MCF-7, NCI-H460, and Hep-G2 cell lines with IC_50_ values of 0.24–0.51 and 0.71–2.42 μM, respectively (Saha et al., 2017). Cultivation of *Streptomyces* sp. OUCMDZ-3434 (*Enteromorpha prolifera*, Zhanqiao Beach, Qingdao, China) produced two new polyketides bearing duble 6-(2-phenylnaphthalene-1-yl)pyrane-2-one nuclei and a methylene linkage, wailupemycins H (**7**) and I (**8**), both of which showed α-glucosidase inhibitority activities with Ki/IC_50_ values of 16.8/19.7 and 6.0/8.3 μM, respectively (Chen et al., 2016g). Neoansamycins A–C (**9**-**11**), three novel naphthalenic octaketide ansamycins with unprecedented n-pentylmalonyl-CoA or n-butylmalonyl-CoA extender units, were produced by *Streptomyces* sp. LZ35 and resulted from activation of a cryptic ansamycin biosynthetic gene cluster (*nam*) (Li et al., 2015f). The first sulfur angucyclinone with an unusual ether-bridged system, grisemycin (**12**), which exhibited a novel cage-like structure, was obtained from the marine sediment-derived *Streptomyces griseus* M268 (Kiaochow Bay, China) (Xie et al., 2016b). Nahuoic acids B–E (**13**-**16**), polyol polyketides with a decalin ring, were produced by the *Streptomyces* sp. SCSGAA 0027 (*Melitodes squamata*, South China Sea) (Nong et al., 2016). Naquihexcins A (**17**) and B (**18**), s-bridged pyranonaphthoquinone dimers with a rare unsaturated hexuronic acid moiety, were isolated from a culture of the sponge-derived *Streptomyces* sp. HDN-10-293 (Che et al., 2016).

#### Mangrove-derived bacteria

The culture of mangrove soil-derived *Streptomyces* sp. 219807 (Hainan, China) afforded eight elaiophylin derivatives including halichoblelide D (**19**), which showed significant cytotoxic activity toward MCF-7 and HeLa cells with the IC_50_ values of 0.33 and 0.30 μM, respectively (Han et al., 2016b). Six new polyene-polyol macrolides, reedsmycins A–F, were afforded by the reed rhizosphere soil-derived *Streptomyces* sp. CHQ-64 (Guangdong, China), among which reedsmycin F (**20**) possessed a rare 31-membered macrocyclic system containing a tetrahydrofuran motif (Che et al., 2015).

### Marine-derived fungi (including mangrove-derived fungi)

#### Marine-derived fungi (except mangrove-derived fungi)

Curindolizine (**21**), an indolizine alkaloid with an unprecedented skeleton, was obtained from the white croaker-associated fungus *Curvularia* sp. IFB-Z10 and displayed anti-inflammatory action in lipopolysaccharide (LPS)-induced RAW 264.7 macrophages with an IC_50_ value of 5.31 μM (positive control dexamethasone, 2.17 μM) (Han et al., 2016c). Acremeremophilanes A–O, 15 new eremophilane-type sesquiterpenoids, were isolated from a deep-sea sediment-derived *Acremonium* sp. (South Atlantic Ocean), among which acremeremophilanes B–F (**22**-**26**) contained a novel 4-hexenoic acid unit. Acremeremophilane B (**22**) showed the inhibition on the LPS-induced NO production in RAW 264.7 macrophage cell lines with an IC_50_ value of 8 μM (reference quercetin, 15 μM) (Cheng et al., 2016). The fungus *Aspergillus ochraceopetaliformis* SCSIO 05702 (sediment, Chinese Antarctic station) produced five highly oxygenated α-pyrone merosesquiterpenoids, ochraceopones A–E, along with a new isomer of asteltoxin, isoasteltoxin (**27**). Ochraceopones A–D (**28**-**31**) were new α-pyrone merosesquiterpenoids with a linear tetracyclic carbon skeleton that had not been described previously. Compound **27** inhibited H1N1 and H3N2 influenza viruses with IC_50_ values of 0.23 and 0.66 μM (tamiflu, 16.9 and 18.5 nM), respectively (Wang et al., 2016b). Six new polyketides (**32**-**37**) with an anthraquinone-xanthone basic structure were produced by *Cacospongia scalaris*-derived fungus, *Engyodontium album* LF069 (Limski Fjord, Croatia). Compounds **33**–**37** represented the first examples of a 23,28 seco-beticolin carbon skeleton. Compounds **32** and **33** showed inhibitory activities against the methicillin resistant *Staphylococcus aureus* (MRSA) with IC_50_ at 0.17 and 0.24 μM, respectively, 10-fold stronger than chloramphenicol (Wu et al., 2016). Pleosporalone A (**38**), the first azaphilone derivative with an aromatic A-ring, were produced by the sediment-derived fungus *Pleosporales* sp. CF09-01 (Bohai Sea, China). Compound **38** showed antifungal activity to three plant pathogenic fungi, *Rhizopus oryzae, Botrytis cinerea*, and *Phytophthora capsici*, with minimal inhibitory concentration (MIC) values of 0.78, 0.39, and 0.78 μM, more potent than carbendazim (MIC 1.56, 0.78, and 1.56 μM), respectively (Cao et al., 2016a). Deep sea sediment-derived *Penicillium aculeatum* SD-321 (South China sea) produced three novel phenolic bisabolane sesquiterpenes, peniciaculins A (**39**) and B, and 1-hydroxyboivinianin A. Peniciaculin A (**39**) displayed antibacteria activity to *Micrococcus luteus* and *Vibrio alginolyticus* with MIC values of 1.0 and 2.0 μg/mL, respectively, comparable to chloramphenicol (8.0 and 0.5 μg/mL). Peniciaculin A (**39**) also showed inhibitory activity against *Alternaria brassicae* with an MIC value at 0.5 μg/mL, much better than amphotericin B (32 μg/mL) (Li et al., 2015h).

**Figure 2:**
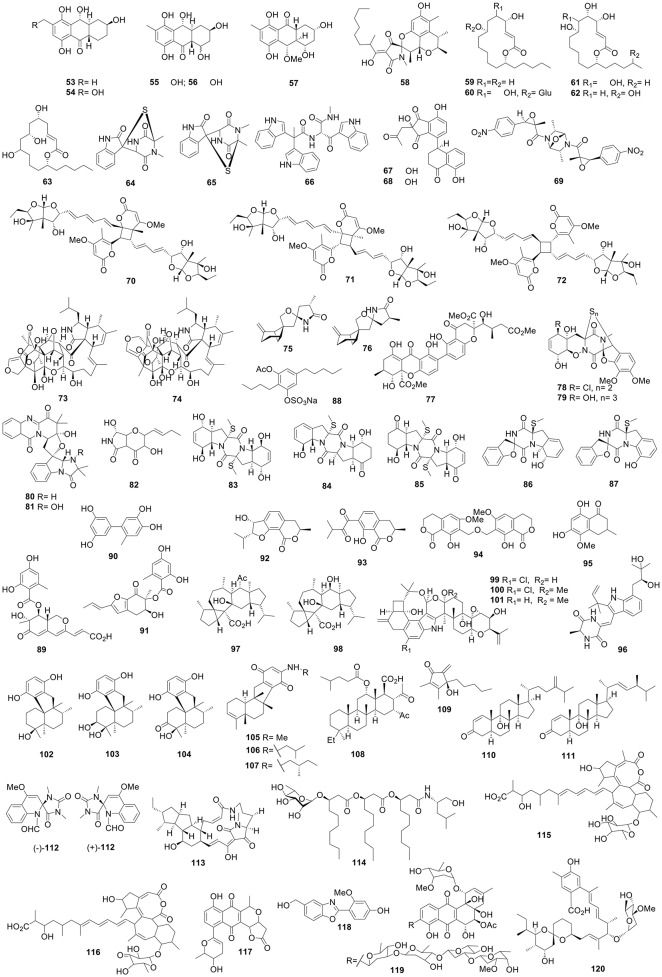
Structures of compounds **53-120**.

New ambuic acid analogs, penicyclones A–E (**40**-**44**), were produced by the deep sea-derived fungus *Penicillium* sp. F23-2 and were found to show antimicrobial activities against *S. aureus* with MICs of 0.3–1.0 μg/mL (Guo et al., 2015a). Adametizines A and B, two novel bisthiodiketopiperazine derivatives, were afforded by the sponge-derived fungus *Penicillium adametzioides* AS-53 (Hainan, China). Adametizine A (**45**) exhibited brine shrimp lethality with a median lethal dose (LD_50_) value of 4.8 μM (colchicine, 8.1 μM) and antimicrobial activity against *S. aureus, Aeromonas hydrophilia*, and *V. parahaemolyticus* with a same MIC value of 8 μg/mL (Liu et al., 2015i). A culture broth of the deep-sea sediment-derived fungus *Spiromastix* sp. MCCC 3A00308 (2869 m, South Atlantic Ocean) contained 11 new polyphenols, spiromastols A–K, among which spiromastols A (**46**) and C (**47**) showed antimicrobial activities against *Agrobacterium tumefaciens* ATCC11158, *Bacillus thuringensis* ATCC10792, *B. subtilis* CMCC63501, *Pseudomonas lachrymans* ATCC11921, *Ralstonia solanacearum* ATCC11696, *S. aureus* ATCC25923, and *Xanthomanes vesicatoria* ATCC11633 with MICs of 0.25–0.5 μg/mL. The MIC range of chloroamphenicol toward the same bacteria was 1.0–2.0 μg/mL (Niu et al., 2015). The marine sponge-derived *Aspergillus* sp. SCSIO XWS02F40 (*Callyspongia* sp, Guangdong, China) yielded asteltoxins E (**48**) and F (**49**), which exhibited anti- virus activities against H3N2 virus with IC_50_ of 6.2 and 8.9 μM, respectively. Asteltoxin E (**48**) also active to H1N1 cirus with the IC_50_ value of 3.5 μM (Tian et al., 2015c). Research into the sponge-derived fungus *Arthrinium arundinis* ZSDS1-F3 (Xisha Islands, China) led to the identification of three novel 4-hydroxy-2-pyridone alkaloids, arthpyrones A–C. Arthpyrone C (**50**) significantly inhibited AchE with an IC_50_ value of 0.81 μM (tacrine, 0.48 μM) (Wang et al., 2015e). The fungus *Pestalotiopsis* ZJ-2009-7-6 associated with *Sarcophyton* sp. (Yongxing Island, China) afforded two new chlorinated enantiomeric diphenylmethanes, (±)-pestalachlorides E (**51**) and F (**52**), both of which showed significant antifouling activities with half maximal effective concentration (EC_50_) values of 1.65 and 0.55 μg/mL, respectively, while SeaNine 211 acted as the positive control with an EC_50_ value of 1.23 μg/mL (Xing et al., 2016). The algicolous fungus *Talaromyces islandicus* EN-501 from *Laurencia okamurai* (Shandong, China) afforded five new polyhydroxylated hydroanthraquinone derivatives (**53**-**57**) (Figure 2), which displayed better antioxidant activities against 2,2-diphenyl-1-picryl-hydrazyl (DPPH) radicals than butylated hydroxytoluene (BHT, 61 μM) with an IC_50_ range of 12–52 μM (Li et al., 2017).

The marine sediment-derived fungus *Penicillium citrinum* (Langqi Island, Fujian, China) afforded penicitrinine A (**58**), an alkaloid with a unique spiro skeleton (Liu et al., 2015d). Five 14-membered macrolides, gliomasolides A–E (**59**-**63**), were isolated from a culture broth of the sponge-associated fungus *Gliomastix* sp. ZSDS1-F7-2 (*Phakellia fusca*, South China Sea) (Zhang et al., 2015h). Fermentation of the fungus *Pseudallescheria ellipsoidea* F42-3 associated with the coral *Lobophytum crassum* (Sanya, Hainan, China) afforded three new compounds (**64**-**66**) with a unique skeleton. Pseudellones A (**64**) and B (**65**) were irregularly bridged epimonothiodiketopiperazine diastereomers with an unusual 3-indolylglycine residue (Liu et al., 2015e). Chrysamides A–C, three dimeric nitrophenyl trans-epoxyamides, were produced by the sediment fungus *Penicillium chrysogenum* SCSIO41001 (Indian Ocean). Chrysamide A (**67**) represented a centrosymmetric dimer skeleton with an novel 7-oxa-2,5-diazabicyclo[2.2.1]heptane nucleus (Chen et al., 2016f). Four new tetralone derivatives, clindanones A (**68**) and B (**69**) along with cladosporols F and G, were afforded by the deep-sea sediment-derived fungus *Cladosporium cladosporioides* HDN14-342 (Indian Ocean), among which compounds **68** and **69** featured new dimeric skeleton coupling indanone and 1-tetralone units (Zhang et al., 2016m). Three novel asteltoxin-bearing dimers, diasteltoxins A–C (**70**-**72**), were produced by a mutated sponge fungus, *Emericella variecolor* XSA-07-2 (Long et al., 2016). Epicochalasines A (**73**) and B (**74**), two new cytochalasans featured a hendecacyclic 5/6/11/5/6/5/6/5/6/6/5 ring system containing fused aspochalasin and epicoccine dimer moieties, were produced by *Aspergillus flavipes* (Zhu et al., 2016). A pair of unusual epimeric spiroaminal derivatives bearing a 6/4/5/5 tetracyclic ring system, sporulaminals A (**75**) and B (**76**), were afforded by the marine sediment-derived fungus *Paraconiothyrium sporulosum* YK-03 (Bohai Bay, China) (Zhang et al., 2016d).

#### Mangrove-derived fungi

Versixanthones A–F, six unusual xanthone-chromanone dimers, were obtained from the mangrove-derived fungus *Aspergillus versicolor* HDN1009 (Guangdong Province, China); versixanthone F (**77**) exhibited cytotoxic activity against HCT-116 with the IC_50_ value of 0.7 μM (Wu et al., 2015a). The mangrove endophytic fungus *Penicillium janthinellum* HDN13-309 (Hainan Province, China) produced six new epipolythiodioxopiperazine (ETP) alkaloids, penicisulfuranols A–F. Penicisulfuranols A (**78**) and C (**79**) displayed cytotoxicities against HeLa and HL-60 cells with IC_50_ values of 0.5/0.1 (HeLa/HL-60) and 0.3/1.2 μM, respectively (Zhu et al., 2017). Endophytic fungus *Neosartorya udagawae* HDN13-313 (*Aricennia marina*, Hainan, China) produced neosartoryadins A (**80**) and B (**81**), which possessed a unique 6/6/6/5 quinazoline ring system directly connected to the 6/5/5 imidazoindolone ring. Neosartoryadins A (**80**) and B (**81**) inhibited H1N1 influenza viruses with IC_50_ values of 66 and 58 μM, respectively, better than ribavirin of 94 μM (Yu et al., 2016a). The mangrove fungus *Penicillium brocae* MA-231 (*Avicennia marina*, Hainan, China) produced one novel derivative of polyoxygenated dihydropyrano[2,3-*c*]pyrrole-4,5-dione, pyranonigrin F (**82**), five new sulfide diketopiperazine derivatives (penicibrocazines A–E), as well as four diketopiperazines (spirobrocazines A–C along with brocazine G). Pyranonigrin F (**82**) displayed potent antimicrobial activity against *S. aureus, Vibrio. harveyi*, and *Vibrio. parahaemolyticus* with the same MIC value of 0.5 μg/mL, more potent than chloromycetin with MICs of 8.0, 2.0, and 128.0 μg/mL, respectively (Meng et al., 2015b). Penicibrocazine C (**83**) inhibited *S. aureus* and *M. luteus* with the same MIC value of 0.25 μg/mL (chloromycetin, 4.0 and 2.0 μg/mL), while both penicibrocazines B (**84**) and E (**85**) inhibited *Gaeumannomyces graminis* with the same MIC value of 0.25 μg/mL (amphotericin B, 16 μg/mL) (Meng et al., 2015c). Spirobrocazines A (**86**) and B (**87**) showed a rare 6/5/6/5/6 cyclic system which had a spirocyclic center at C-2 (Meng et al., 2016a).

A fermentation broth of the mangrove fungus *Stemphylium* sp. 33231 endophytic with *Brguiera sexangula* var. *rhynchopetala* (South China Sea) afforded two novel stemphol sulfates, stemphols A and B. Stemphol B (**88**) showed antimicrobial activities against *Escherichia coli* and *B. cereus* with the same MIC value of 0.6 μg/mL (ciprofloxacin, 0.3 μg/mL) (Zhou et al., 2015d). Endophytic fungus *Penicillium* sp. HN29-3B1 (*Cerbera manghas*, Dongzhaigang, Hainan, China) produced pinazaphilones A and B, penicidone D, and two phenolic compounds. Pinazaphilone B (**89**) and phenolic compound **90** showed more effective α-glucosidase inhibition than acarbose (446.7 μM) with IC_50_ values of 28.0 and 2.2 μM, respectively (Liu et al., 2015j). Aspergifuranone (**91**) was identified from the algicolous fungus *Aspergillus* sp. 16-5B with *Sonneratia apetala* (Dongzhaigang, Hainan, China) and exhibited significant α-glucosidase inhibition with an IC_50_ value of 9.05 μM (acarbose, 553.7 μM) (Liu et al., 2015h). Eight isocoumarin derivatives and one isoquinoline were afforded by the mangrove fungus *Aspergillus* sp. 085242 endophytic with *Acanthus ilicifolius* (Shankou, Guangxi, China). Asperisocoumarins B (**92**), E (**93**), and F (**94**) exhibited α-glucosidase inhibition with IC_50_ values of 87.8, 52.3, and 95.6 μM, respectively (acarbose, 628.3 μM) (Xiao et al., 2016b). Six new isocoumarins and two new benzofurans were produced by the mangrove fungus *Talaromyces amestolkiae* YX1 endophytic with *Kandelia obovate* (Zhanjiang, Guangdong, China), among which new isocoumarin **95** showed better α-glucosidase inhibition than acarbose with IC_50_ value of 89.4 and 958.3 μM, respectively (Chen et al., 2016e). Mangrove rhizospheric soil-derived *Eurotium rubrum* MA-150 (Andaman Sea coastline, Thailand) yielded three new indolediketopiperazine alkaloids, rubrumazines A–C. Among these compounds, rubrumazine B (**96**) exhibited the best brine shrimp lethality with an LD_50_ value of 2.4 μM (colchicine, 19.4 μM) (Meng et al., 2015a).

Two new sesterterpenoids, aspterpenacids A (**97**) and B (**98**), from *Aspergillus terreus* H010, the endophytic fungus of *Kandelia obovata*, represented an unusual 5/3/7/6/5 carbon ring skeleton (Liu et al., 2016e). Twenty indole-diterpenes including three new ones were obtained from a culture of the mangrove fungus *Mucor irregularis* QEN-189 associated with *Rhizophora stylosa*, (Hainan, China). Compounds **99**–**101** possessed a rare 4,6,6,8,5,6,6,6,6-fused indole-diterpene ring system (Gao et al., 2016b).

## Marine animals

A total of 189 new NPs was identified from marine animals in 2015–2016 including eight (4.2%) with novel skeletons and 55 (29.1%) with various bioactivities. Herein, we list six references that report 11 NPs with a novel skeleton or/and significant bioactivity.

Four new 6/6/5/6-fused tetracyclic meroterpenes, dysiherbols A–C (**102**-**104**) and dysideanone E, were obtained from a marine sponge *Dysidea* sp. (South China Sea, at a depth of 10 m). Dysiherbol A (**102**) showed potent nuclear factor-kappaB (NF-κB) inhibition and cytotoxicity with IC_50_ values of 0.49 and 0.58 μM, respectively (Jiao et al., 2016). *Dysidea fragilis* (South China Sea) was also the source of three new sesquiterpene aminoquinones ferturing the rearranged avarone skeleton, dysifragilones A–C (**105**-**107**), which inhibited the production of NO stimulated by LPS in mouse RAW 264.7 macrophages with IC_50_ values of 6.61, 9.83, and 17.22 μM, respectively (positive control hydrocortisone, 45.72 μM) (Jiao et al., 2015a). Eight novel scalarane sesterterpenoids, carteriofenones D–K, were discovered from the marine sponge *Carteriospongia foliascens* (Dongluoxigu Island, China). Carteriofenone D (**108**) was cytotoxic to a mouse lymphocytic leukemia cell line (P388) with an IC_50_ value of 0.96 μM (Cao et al., 2015a). Five novel metabolites were isolated from the soft coral *Sinularia verruca* (Ximao Island, Hainan, China), among which compound **109** showed anti-human immunodeficiency virus (HIV)-1 activity with an EC_50_ of 5.8 μM (Yuan et al., 2016). Investigation of the gorgonian coral *Subergorgia rubra* (South China sea) led to the isolation of three new Δ^1^-9-hydroxy-3-ketosteroids, subergosterones A–C. Subergosterones B (**110**) and C (**111**) exhibited antibacterial activities to *B. cereus* with an MIC value of 1.56 μM, similar to that of ciprofloxacin (1.25 μM) (Sun et al., 2015c). A pair of novel bisheterocyclic quinolineimidazole alkaloids, (±)-spiroreticulatine ((±)-**112**), obtained from sponge *Fascaplysinopsis reticulata* (South China Sea), were the first example of spiro quinoline-imidazole alkaloids from sponge (Wang et al., 2015i).

## Terrestrial microorganisms

A total of 1,081 new NPs was produced by terrestrial microorganisms, of which 970 were produced by fungi. Among the new compounds isolated from fungi, more than 7.9% possessed an unprecedented skeleton. All 158 NPs with novel skeletons or/and significant bioactivities are listed.

### Terrestrial-sourced bacteria

**Figure 3:**
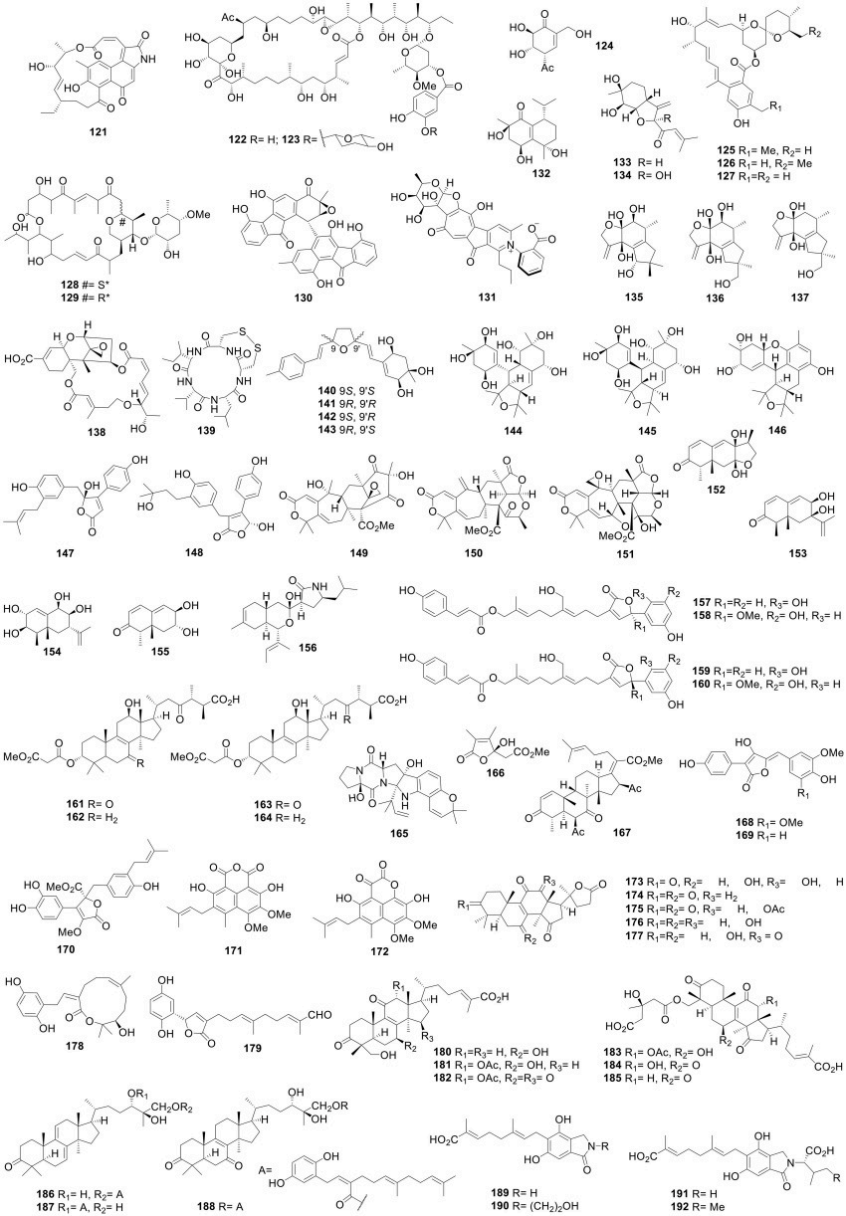
Structures of compounds **121-192**.

A culture of *Lysobacter enzymogenes* (University of Nebraska Lincoln, U.S.) afforded two novel PTMs, lysobacteramides A and B. Lysobacteramide B (**113**) was cytotoxic to A549 cells with an IC_50_ of 0.8 μM (Xu et al., 2015b). Rhizoleucinoside (**114**) with a trimeric 3-hydroxy heptylate nucleus, was obtained from a culture of the rhizobial *Bradyrhizobium* sp. BTAi1 (ATCC BAA-1182) (Chen et al., 2016c). Three polyene antibiotics, aurantinins B–D, were obtained from the fermentation of *Bacillus subtilis* fmb60 (Jiangsu, China). Aurantinins C (**115**) and D (**116**) showed antibacterial activity against *Clostridium sporogenes* with a MIC of 0.78 μg/mL (erythromycin gluceptate, 3.12 μg/mL) (Yang et al., 2016f). The karst cave soil-derived *Streptomyces* sp. CC8-201 (Chongqing, China) produced a new pyranonaphthoquinone (PNQ) antibiotic, xiakemycin A (**117**), which possessed cytotoxicity against HeLa, HCT-116, SH-SY5Y, and PC-3 cells with IC_50_ values of 0.43–0.98 μM (Jiang et al., 2015b). The saltmarsh soil-derived *Nocardiopsis lucentensis* DSM 44048 produced seven new benzoxazole derivatives, nocarbenzoxazoles A–G. Nocarbenzoxazole G (**118**) displayed selective cytotoxicity against HeLa cells with an IC_50_ of 1 μM (Sun et al., 2015a). Chattamycins A and B, two novel angucycline antibiotics, were produced by *Streptomyces chattanoogensis* L10 (CGMCC 2644). Chattamycin B (**119**) showed stronger cytotoxicity against MCF-7 cells with an IC_50_ of 1.08 μM when compared with the positive control (Zhou et al., 2015e). A culture of the Δ*ave*CDE mutant strain *Streptomyces avermectinius* afforded three novel 1,19-seco-avermectin (AVE) analogs, among which compound **120** (Figure 3) was cytotoxic against the Saos-2 cells with an IC_50_ value of 0.7 μM (Sun et al., 2015b). The *hgc1*-overexpressed mutant strain *Streptomyces* sp. LZ35 produced three new hygrocins, among which hygrocin H (**121**) displayed significant cytotoxic activity against HeLa cells with an IC_50_ of 0.8 μM (Li et al., 2015g).

Soil-derived *Streptomyces phytohabitans* HBERC-20821 from Wawushan Hill (Sichuan Province, China) afforded two novel 32-membered macrolides, novonestmycins A (**122**) and B (**123**), both of which were mycostatic against the phytophathogenic fungi *Septoria nodorum, Corynespora cassiicola*, and *Rhizoctonia solani* with MIC values of 0.78, 0.78, and 0.39 μg/mL, respectively. Compounds **122** and **123** were also cytotoxic against HepG2, MCF-7, HeLa, BGC-823, and BEL-7402/5-FU cells with IC_50_ values of 0.15–0.48 and 0.24–1.34 μg/mL, respectively (Wan et al., 2015). Two new polyoxygenated cyclohexenone, gabosines P (**124**) and Q, together with two known cyclic dipeptides were obtained from a culture of *Streptomycetes* strain no. 8 (Qinling Mountains, Shaanxi, China). Gabosine P (**124**) showed α-glucosidase inhibition with an IC_50_ value of 9.07 μM, more potent than acarbose (663.28 μM) (Wei et al., 2016a). 13α-Hydroxy-4-ethyl milbemycin β*3* (**125**), 13α-hydroxy-25-ethylmilbemycin β*3* (**126**), and 13α-hydroxymilbemycin β*3* (**127**), three novel β-class milbemycins, were obtained from a fermentation broth of the genetically engineered strain *Streptomyces avermitilis* MHJ1011. The *aveA1* gene was seamlessly replaced by the *milA1* gene, and compounds **125**-**127** showed significant acaricidal activity with LC_50_ values of 0.0210, 0.1023, and 0.1090 mg/L, respectively (milbemycins A3/A4, 0.0324 mg/L; tenvermectins A/B, 0.0050 mg/L) (Pan et al., 2016a). Two new macrolides with a tetrahydropyran moiety, FXJ15321 (**128**) and FXJ15322 (**129**), were obtained from a fermentation broth of the red soil-derived *Streptomyces* sp. FXJ1.532 (Jiangxi, China) (Guo et al., 2015b). Culture of *Streptomyces coelicolor* YF11 with a heterologous expression of the intact *fls*-gene cluster in a 3% sea salt medium led to the isolation of an unusual heterodimer, difluostatin A (**130**) (Yang et al., 2015a). Rubrolone B (**131**), a new class of rubrolone analog possessing a rare benzoic acid-pyridine inner salt moiety, was afforded by *Camellia sinensis*-derived *Streptomyces* sp. KIB-H033 (Yan et al., 2016).

### Terrestrial-sourced fungi

The endophytic fungus *Aspergillus clavatus* from the leaves of *Tripterygium hypoglaucum* yielded a new cadinane-type sesquiterpenoid, aspergillusone D (**132**), which exhibited cytotoxicity against the A549 cells with an IC_50_ value of 0.2 μM (Wang et al., 2015b). Two new bisabolane-type sesquiterpenoids, pleurotons A (**133**) and B (**134**), along with three clitocybulol derivatives, clitocybulols D–F (**135**-**137**) were produced by the edible fungus *Pleurotus cystidiosus* (Qingyun mountains, Fujian, China). These compounds possessed significant inhibitory activities against two human prostate cancer cells, DU-145 and C42B, with IC_50_ values of 28–233 and 52–163 nM, respectively (Zheng et al., 2015b). Epiroridin acid (**138**), a roridin-type trichothecene macrolide, was obtained from the liquid culture of *Myrothecium roridum* A553 associated with *Pogostemon cablin* (Guangdong, China), and showed significant cytotoxicity against MCF-7, SF-268, NCI-H460, and HepG-2 cells with IC_50_ values of 0.170, 0.751, 0.360, and 0.380 μM, respectively (Liu et al., 2016c).

Research into the fungus FR02 endophytic with roots of *Ficus carica* (Qinling Mountain, Shaanxi, China) led to the discovery of 14-membered cyclic dipeptides, among which malformin E (**139**) showed cytotoxicity against MCF-7 and A549 cell lines with IC_50_ values of 0.65 and 2.42 μM, respectively. Meanwhile, malformin E (**139**) also displayed potent antibacterial activity against *B. subtilis* with an MIC value of 0.91 μM (gentamicin, 4.06 μM) (Ma et al., 2016d). Seven dimeric acremines, bisacremines A–D (**140**-**143**) (Wu et al., 2015d) and bisacremines E–G (**144-146**) (Wu et al., 2015e), were produced by the soil-derived *Acremonium persicinum* SC0105 (Dinghu Mountain, Guangdong, China). Bisacremine G (**146**) inhibited the LPS-stimulated production of TNF-α, IL-6, and NO in macrophage and NO in macrophages with 80.1, 89.4, and 55.7% inhibitions, respectively at 50 μM (dexamethasone with 78.0, 92.6, and 62.6% inhibition, respectively). The endophytic fungus *Aspergillus terreus* PR-P-2 from *Camellia sinensis* var. *assamica* (Yunnan, China) yielded three new butenolides, asperteretals A–C. Asperteretals A (**147**) and C (**148**) inhibited the NO production induced by LPS in RAW 264.7 macrophages with IC_50_ values of 26.64 and 17.21 μM, respectively (hydrocortisone, 48.66 μM) (Guo et al., 2016). Five new meroterpenoids, purpurogenolides A–E, were obtained from a solid culture of the soil-derived *Penicillium purpurogenum* MHz 111 (Heilongjiang, China). Among them, purpurogenolides B–D (**149**-**151**) showed inhibitory activities against NO production in LPS-activated BV-2 microglial cells with IC_50_ values of 15.5, 8.8, and 0.8 μM, respectively (indomethacin, 34.5 μM) (Sun et al., 2016b).

The endophytic fungus *Periconia* sp. F-31 with *Annona muricata* (Hainan, China) yielded nine new polyoxygenated eremophilane sesquiterpenes, periconianones C–K and other four new polyketide synthase-nonribosomal peptide synthetase (PKS-NRPS) hybrid metabolites, pericoannosins A–D. Periconianone C (**152**) was the first furan-type isoeremophilane containing a C-8/C-11 linkage and a 7,12-epoxy moiety (Liu et al., 2016d), while pericoannosin A (**156**) was characterized by a novel hexahydro-1*H*-isochromen-5-isobutylpyrrolidin-2-one skeleton (Zhang et al., 2015b). Periconianones D (**153**), G (**154**), and K (**155**) inhibited NO production in LPS-activated BV-2 microglial cells by 10.2, 18.3, and 16.1% inhibition, respectively at 1.0 μM (curcumin 12.9%) (Liu et al., 2016d). Ganosinensols A–D (**157**-**160**), four new farnesyl phenolic compounds, were obtained from the fruiting bodies of *Ganoderma sinense*, and inhibited LPS-induced NO production in RAW 264.7 macrophages with IC_50_ values of 1.15–2.26 μM (hydrocortisone, 58.79 μM) (Wang et al., 2016d). Eight 24-methyllanostane triterpenes, officimalonic acids A–H together with one known lanostane triterpene were obtained from the fruiting bodies of *Fomes officinalis* (Xinjiang, China). Officimalonic acids D (**161**), E (**162**), G (**163**), and H (**164**) were active against NO production in LPS-stimulated RAW 264.7 cells with IC_50_ values of 5.1–8.9 μM (dexamethasone 22.2 μM) (Han et al., 2016a).

Prenylated indole alkaloid speramides A and B, were produced by the fresh water-derived fungus *Aspergillus ochraceus* KM007 (Lake Fuxian, Yunnan, China). Speramide A (**165**) displayed antibacterial activity against *P. aeruginosa* with an MIC value of 0.8 μM (Chang et al., 2016). A new furanone (**166**) was obtained from the edible mushroom *Grifola frondosa* (Wuyi Mountain, Fujian, China), and exhibited antifungal activity against the human pathogen *Pseudallescheria boydii* (amphotericin B, 0.31 μg/mL) as well as the plant pathogens *Piricularia oryzae, Fusarium oxysporum*, and *Gibberella zeae* (carbendazim, 5, 10, and 2.5 μg/ mL) with MIC values of 0.15, 1.25, 2.5, and 2.5 μg/mL, respectively (He et al., 2016). A culture of the endophytic fungus *Fusarium* sp. from the leaves of *Ficus carica* (Qinling Mountain, Shaanxi, China) afforded a new helvolic acid derivative **167**, which displayed activity against some Gram positive and negative (G^+^/G^−^) bacteria and plant pathogenic fungi with MIC values of 3.13–25 μg/mL, more potent than streptomycin sulfate (MIC 7.8 μg/mL for bacteria) and penicillin and carbendazim (MIC 31.2 and 62.5 μg/mL for fungi, respectively) (Liang et al., 2016).

The wetland mud-derived fungus *Aspergillus flavipes* PJ03-11 (Red Beach National Nature Reserve, Liaoning, China) produced three novel butenolides (Zhang et al., 2016f) and three new phenalenone derivatives, flaviphenalenones A–C (Zhang et al., 2016e). Aspulvinones P (**168**), Q (**169**), and methybutyrolactone III (**170**), as well as flaviphenalenones B (**171**) and C (**172**) showed more potent α-glucosidase inhibitory activities than acarbose (0.685 mM) with IC_50_ values of 0.079, 0022, 0.016, 0.095, and 0.079 mM, respectively. The fruiting bodies of *Ganoderma lucidum* (Kingsci Biotechnology Co. Ltd., China) afforded 12 highly oxygenated lanostane triterpenoids, among which compounds **173-177** were nor-lanostanoids containing a 17β-pentatomic lactone ring. Compounds **173**, **177** and verapamil could increase the adriamycin (ADM) accumulation in MCF-7/ADR cells ~3-fold at a concentration of 20 μM when compared with the negative control. Furthermore, compounds **174**, **176**, and **177** showed α-glucosidase inhibition with IC_50_ values of 81.8, 41.7, and 91.3 μM (acarbose, 669.7 μM) (Zhao et al., 2015c).

**Figure 4:**
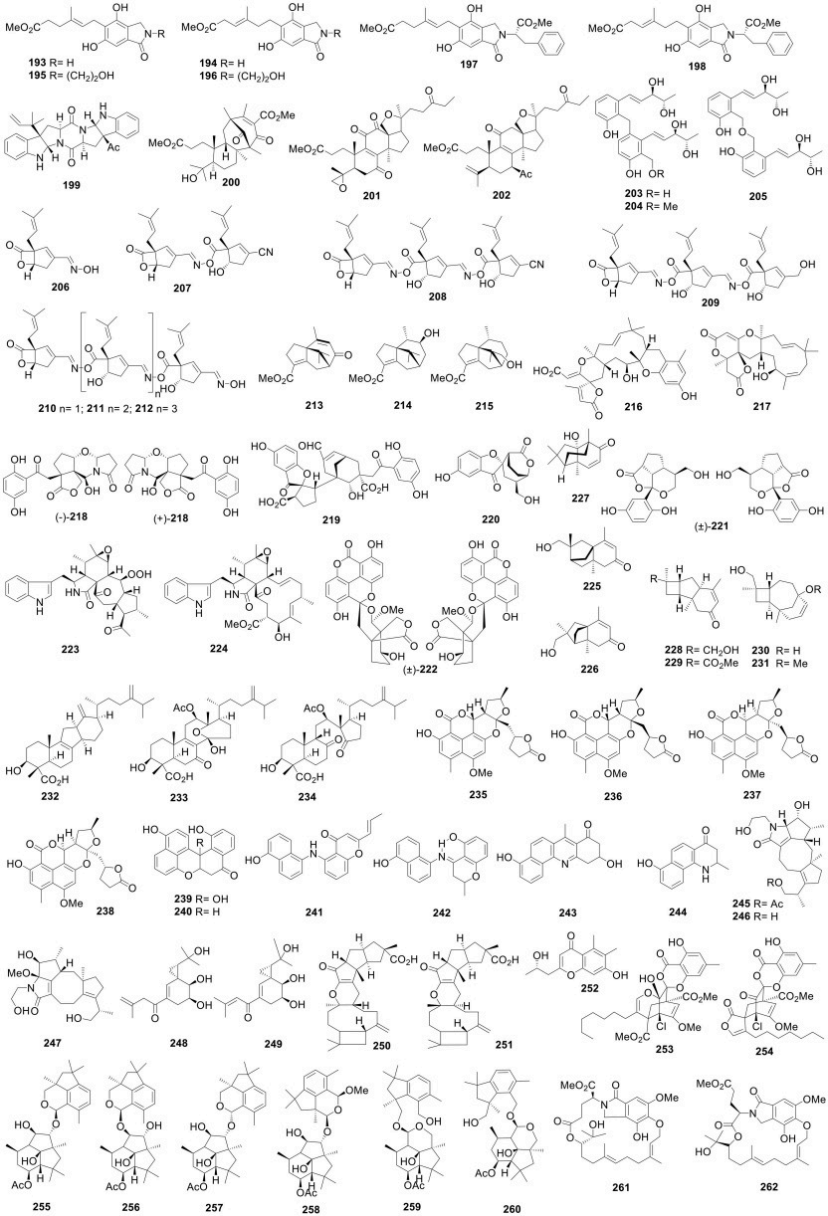
Structures of compounds **193-262**.

Meroterpenoids ganoleucins A (**178**) and C (**179**) were afforded by the fruiting bodies of *Ganoderma leucocontextum* (Nyingchi, Tibet, China) and exhibited stronger α-glucuronidase inhibition than acarbose with IC_50_ values of 6.3, 12.7, and 273 μM (Wang et al., 2016c). Sixteen new lanostane triterpenes, ganoleucoins A–P, were also produced by a *Ganoderma leucocontextum*. Ganoleucoins A (**180**), C (**181**), F (**182**), and J–N (**183**-**187**) showed stronger HMG-CoA reductase inhibition than atorvastatin (IC_50_, 32.1 μM) with IC_50_ values of 10.7–26.6 μM. Ganoleucoins M (**186**), N (**187**), and P (**188**) showed potent α-glucosidase inhibition with IC_50_ values of 13.6, 2.5, and 5.9 μM in contrast with acarbose (273.1 μM) (Wang et al., 2015g). Solid-state fermentation the mushroom *Hericium erinaceus* (Tibet, China) produced 10 new isoindolin-1-ones, erinacerins C–L (**189**-**198**) (Figure 4), most of which possessed α-glucosidase inhibition with IC_50_ values of 5.3–145.1 μM (acarbose, 382.7 μM) (Wang et al., 2015f). *Aspergillus terreus* CGMCC 3.05358 produced amauromine B (**199**), a novel diketopiperazine alkaloid which displayed more potent α-glucosidase inhibition than reference acarbose with IC_50_ values of 0.30 and 0.66 mM, respectively (Shan et al., 2015). Asperterpene B (**200**) possessing a noval 1,2,5-trimethyl-4,9-dioxobicyclo[3.3.1]non-2-ene-3-carboxylic acid moiety, was produced by soil-derived *A. terreus* (Yangzi River, Hubei, China), and was a growth inhibitor of BACE1 with an IC_50_ of 59 nM (positive control LY2811376, 260 nM) (Qi et al., 2016).

The fruiting bodies of *Ganoderma boninense* (Hainan, China) contained ganoboninones A–F, six new 3,4-seco-27-norlanostane triterpenes. Ganoboninones B (**201**) and F (**202**) showed *in vitro* antimalarial activities against *Plasmodium falciparum* with IC_50_ values of 15.68 and 2.03 μM, more potent than artemisinin (18.61 μM) (Ma et al., 2015c). Three novel sordariol dimmers **203**–**205** were afforded by plant fungus *Sordaria macrospora* from *Ilex cornuta* and showed antioxidation against 2,2-azino-bis-3-ethylbenzothiazoline-6-sulfonic acid (ABTS) radical with EC_50_ values of 12.2–14.5 μM (reference trolox, 26.7 μM) (Li et al., 2016h). Research into *Boreostereum vibrans* (Kunming Botanical Garden, China) led to the isolation of 16 new oximes and oxime esters. Vibralactoximes A (**206**), D (**207**), E (**208**), G (**209**), and I–K (**210**-**212**) displayed significant pancreatic lipase inhibitory activities with IC_50_ values of 11.1–28.6 μM (vibralactone, 48.7 μM) (Chen et al., 2016b). Microbial transformation of methyl cyperenoate by *Cunninghamella elegans* CGMCC 3.2028 led to the isolation of eight new derivatives, among which compounds **213**-**215** displayed *in vitro* antiplatelet aggregation activities with 93.01, 96.37, and 82.68% inhibition, respectively at 400 μg/mL, equivalent to aspirin (86.32%) (Tian et al., 2016b). Phomanolides A (**216**) and B (**217**), unique meroterpenoids with new pentacyclic and tetracyclic skeletons, respectively, were isolated from the solid cultures of the soil-derived *Phoma* sp. (Qinghai-Tibetan plateau, China) (Zhang et al., 2015i). The novel hybrid metabolite (±)-sinensilactam A ((±)-**218**) was isolated from the fruit bodies of *Ganoderma sinensis*, and possessed a unique 2*H*-pyrrolo[2,1-b][1,3]oxazin-6(7*H*)-one ring system (Luo et al., 2015b).

Culture of *Ganoderma applanatum* produced applanatumin A (**219**) with a new hexacyclic skeleton bearing a spiro[benzo furan-2,1′-cyclopentane] motif (Luo et al., 2015a). applanatumol A (**220**) with a new spiro[benzofuran-2,2′-bicyclo[3.2.2]nonane] ring system and (±)-applanatumol B ((±)-**221**) with an unusual dioxacyclopenta[*cd*]inden motif (Luo et al., 2016a), as well as (±)-ganoapplanin ((±)-**222**) with a dioxaspirocyclic skeleton constructed from a 6/6/6/6 tetracyclic system and a tricyclo[4.3.3.0^3^′, 7′]dodecane motif (Li et al., 2016f). Armochaeglobines A (**223**) and B (**224**), two cytochalasan alkaloids, were produced by the insect-derived *Chaetomium globosum* TW1-1 associated with *Armadillidium vulgare*. Armochaeglobine A (**223**) possessed an unprecedented tetracyclic 5/6/7/5 system, while armochaeglobine B (**224**) possessed a rare 12-membered cyclic carbon scaffold (Chen et al., 2015a). Polycyclic compounds with unusual skeletons (**225**-**231**) were produced by the endophytic *Aspergillus tubingensis* KJ-9 through remodeling of the β-caryophyllene skeleton, among which compound **225** featured a novel 5/5/6 sesquiterpene skeleton (Tang et al., 2015). Ten new ergosteroids, gloeophyllins A–J, were isolated from the solid cultures of *Gloeophyllum abietinum*. Gloeophyllin A (**232**) had a rare C-nor-D-homosteroid skeleton, while gloeophyllin I (**233**) possessed an unprecedented ergostane skeleton with the 6/5 fused C/D rings replacing by a 10-oxabicyclo[4.3.1]decane moiety, and gloeophyllin C (**234**) represented the first example of ergosteroid that featured the cleavage of a C8–C14 bond (Han et al., 2015). Four new oxaphenalenone ketals, neonectrolides B–E (**235**-**238**), incorporating the furo[2,3-*b*]isochromeno [3,4,5-*def*] chromen-11(6a*H*)-one skeleton, were produced by the soil-derived *Neonectria* sp. (Qinghai-Tibetan plateau, China) (Ren et al., 2015b). Mutadalesols A–F (**239**-**244**), new naphthalene-based molecules, were mycosynthesized by using the Δ*pksTL* mutant strain of insect-derived *Daldinia eschscholzii* from *Tenodora aridifolia* (Tian et al., 2015b). Diterpenoid alkaloids, pericolactines A–C (**245**-**247**) with an unusual 5/5/8/5 tetracyclic system, were afforded by *Periconia* sp. (Changbai Mountain, Jilin, China) (Wu et al., 2015c). Pestalotriols A (**248**) and B (**249**), featuring an unprecedented spiro[2.5]octane skeleton, were afforded by *Pestalotiopsis fici* endophytic with *C. sinensis* (Zhejiang, China) (Liu et al., 2015c).

**Figure 5:**
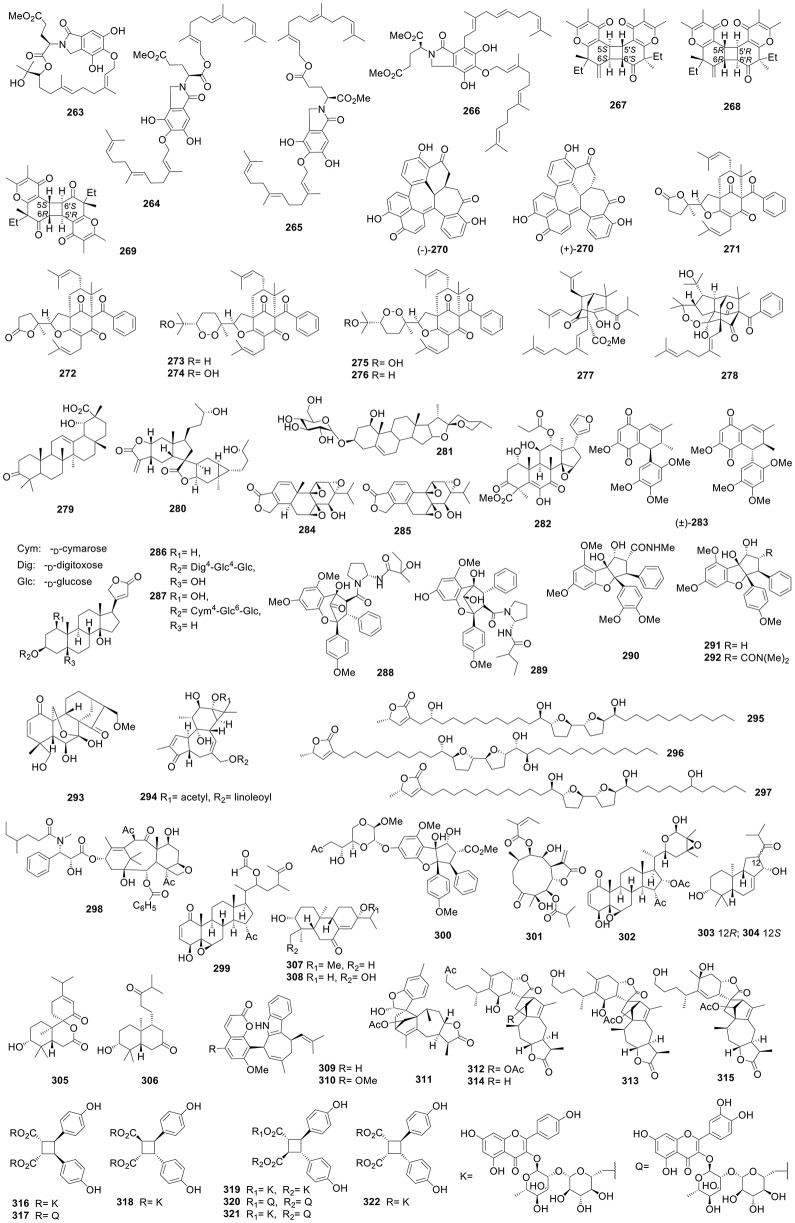
Structures of compounds **263-322**.

Heterodimeric sesquiterpenes, sterhirsutins C (**250**) and D (**251**), together with sesquiterpenoids, sterhirsutins E–L, were produced by *Stereum hirsutum*. Sterhirsutins C (**250**) and D (**251**) possessed an unusual 5/5/5/6/9/4 fused ring system (Qi et al., 2015). Four new chromones, chaetosemins B–E, along with chaetosemin A and *S*(+)-chaetoquadrin J, were isolated from a solid culture of *Chaetomium seminudum* (Shaanxi, China), among which chaetosemin D (**252**) possessed a new skeleton (Li et al., 2015b). Two new spiroketals with an unique [4,7]methanochromene and dispirotrione skeleton, chlorotheolides A (**253**) and B (**254**), were isolated from a solid culture of *Pestalotiopsis theae* N635 associated with *C. sinensis* (Zhejiang, China) (Liu et al., 2016f). Six new heterodimeric botryane ethers, hypocriols A–F (**255**-**260**), were produced by the insect-derived *Hypocrea* sp. EC1-35 (*Septobasidium*-infected *Serrataspis* sp.) (Ren et al., 2016). The endophytic *Emericella nidulans* HDN12-249 (Laizhou Bay, China) produced six isoindolones, emericellolides A–C (**261**-**263**) (Figure 5) and emeriphenolicins E–G (**264**-**266**) (Zhou et al., 2016a). Three dimeric spiciferones with an acyclobutane ring, lecanicillones A–C (**267**-**269**), were afforded by the entomopathogenic *Lecanicillium* sp. PR-M-3 (Wang et al., 2016h). Culture of the insect-associated *Daldinia eschscholzii* IFB-TL01 from *Tenodera aridifolia* led to the isolation of selesconol ((±)-**270**) that could induce the differentiation of rat bone marrow mesenchymal stem cells into neural cells (Zhang et al., 2016a).

## Terrestrial plants

Terrestrial plants afforded 4,875 new NPs in the past 2 years and undoubtedly, are the most irreplaceable source of novel NPs in China. Compounds with unique skeletons and bioactivities are also mainly from terrestrial plants, although the numbers of both are lower than average. A total of 451 new NPs with novel skeletons or/and significant bioactivities are listed here.

Hyperisampsins H–M (**271**-**276**) were new polycyclic polyprenylated acylphloroglucinols (PPAPs) obtained from the aerial parts of *Hypericum sampsonii* (Dabie Mountain, Hubei, China). Hyperisampsins H (**271**) and I (**272**) represented the first examples of PPAPs featuring an unusual γ-lactone ring at C-23, while hyperisampsins J–M (**273**-**276**) had an 1,2-dioxane ring. Hyperisampsin J (**273**) exhibited significant cytotoxic activity to HL-60, SMMC-7721, A-549, MCF-7, and SW480 cell lines with the IC_50_ range of 0.56–2.49 μM (reference DDP, 1.17–15.86 μM) (Zhu et al., 2015a). Another two PPAPs, hypersubones A (**277**) and B (**278**), were also obtained by culture of *Hypericum subsessile*. Hypersubone A (**277**) had a new *seco*-adamantane skeleton, while hypersubone B (**278**) had a tetracyclo-[6.3.1.1.0]-tridecane core fused in a peroxide ring. Hypersubone B (**278**) was cytotoxic to HepG2 and Eca109 cells with IC_50_ values of 1.58 and 0.07 μM (Liao et al., 2015b). A new triterpenoid (**279**) was isolated from *Maytenus austroyunnanensis* (Yunnan, China) and displayed inhibitory activity against HeLa cells with an IC_50_ of 1.48 μM (Tan et al., 2016). Isolation of whole plants of *Carpesium abrotanoides* gave a novel dimeric sesquiterpene with a cyclopentane linker, dicarabrol (**280**). Dicarabrol (**280**) was active against SGC-7901, U937, HeLa, DU145, and HL-60 cells with the IC_50_ range of 0.10–0.71 μM. Dicarabrol (**280**) was also antimycobacterial with an MIC of 3.7 μM, close to controlisoniazid (2.0 μM) (Wang et al., 2015d). Isolation of fresh rhizomes of *Tupistra chinensis* (Shennongjia, Hubei, China) afforded three new spirostanol saponins, tupistrosides G–I, and one new flavane-*O*-glucoside, tupichiside A. Tupistroside H (**281**) showed significant cytotoxicity against LoVo and BGC-823 cell lines with IC_50_ values of 0.267 and 0.327 μM, respectively, in contrast to that of 5.92 and 4.59 μM for cisplatin (Xiao et al., 2015). The leaves of *Trichilia americana* (Mengla, Yunnan, China) contained 10 novel cedrelone limonoids, among which compound **282** exhibited cytotoxic activity against MCF-7, SMMC-7721, HL-60, A-549, and SW480 cells with IC_50_ values ranging from 1.0 to 3.1 μM (Ji et al., 2015). A pair of novel quinone enantiomers, (±)-merrilliaquinone ((±)-**283**), was obtained from the branches and leaves of *Illicium merrillianum* (Gongshan, Yunnan, China). Compound (+)-**283** selectively inhibited SMMC7721 and HuH7 cells with IC_50_ values of 0.91 and 1.29 μM in contrast with the CC_50_ of QSG7701 and L02 cells at 47.79 and 36.71 μM, respectively (Tian et al., 2015a).

The leaves of *Tripterygium wilfordii* (Taining, Fujian, China) contained six new abietane diterpenoids, tripterlides A–F. Tripterlides E (**284**) and F (**285**) displayed potent cytotoxicities against HCT-116, HepG2, BGC-823, and H460 cells with IC_50_ ranges of 0.93–3.16 and 0.17–0.90 μM, respectively (Wang et al., 2015a). Six new cardenolide glycosides were discovered from the roots of *Streptocaulon juventas* (Yunnan, China). Compounds **286** and **287** showed significant activities to A549 cells with IC_50_ values of 0.016 and 0.38 μM, respectively (Ye et al., 2015). The leaves of *Aglaia odorata* (Xishuangbanna, Yunnan, China) afforded nine novel flavaglines, aglaodoratins A–I. Aglaodoratin C (**288**) exhibited cytotoxicity against MG-63 and HT-29 cells with IC_50_ values of 1.2 and 0.097 μM, and aglaodoratin D (**289**) was cytotoxic against the MG-63 cells with an IC_50_ of 0.75 μM (An et al., 2015). The twigs of *Aglaia odorata* (Longzhou, Guangxi, China) afforded compound **290**, which was active against SGC-7901, HeLa, and A-549 cells with IC_50_ values of 0.12, 0.32, and 0.25 μM, respectively (Peng et al., 2016b). The roots of *Aglaia odorata* contained rocaglaol (**291**) and rocaglamide (**292**), both were active against MCF-7, SMMC-7721, HL-60, A-549, and SW480 cells with IC_50_ ranges of 0.007–0.095 μM (Liu and Xu, 2016). Neolaxiflorins I–Y, 17 novel *ent*-kaurane diterpenoids, were obtained from the *Isodon eriocalyx* var. *laxiflora* leaves (Yunnan, China), among which neolaxiflorin P (**293**) displayed the best activity against A-549, HL-60, MCF-7, SMMC-7721, and SW-480 cells with IC_50_ ranges of 0.45–1.12 μM (Wang et al., 2015j).

Research into the seeds of *Croton tiglium* (Sichuan, China) afforded four new 4-deoxy-4β-phorbol diesters. Compound **294** was cytotoxic toward SNU387 cell line with an IC_50_ value of 0.71 μM (Zhang et al., 2016j). The seeds of *Annona squamosa* (Guangdong, China) afforded four new annonaceous acetogenins (ACGs), squamocins I–III (**295**-**297**), and squamoxinone D. Compound **297** were most active against H460 cell line with an IC_50_ value of 0.0492 μg/mL (Miao et al., 2016). Four new taxane derivatives were obtained from the whole plants of *Taxus wallichiana*. var. *mairer* (Jiangsu, China); compound **298** was cytotoxic against the MCF-7 cell line with an IC_50_ of 0.077 μM (Wang et al., 2016f). Six new withanolides and four new withanolide glucosides were obtained from *Physalis pubescens* (Liaoning, China). Compounds **299** and **300** were active against C4-2B, CWR22Rvl, 786-O, A-498, Caki-2, ACHN, A375, and L02 cell lines with IC_50_ values ranging from 0.17 to 1.22 μM (Xia et al., 2016). Aglapervirisin A (**301**) was isolated from the leaves of *Aglaia perviridis* (Xishuangbanna, Yunnan, China), and showed cytotoxicity against HepG2, HL-60, MCF-7, and HT-29 cell lines with IC_50_ values from 0.008 to 0.014 μM (An et al., 2016b). Whole plants of *Carpesium cernuum* (Guizhou, China) contained 10 novel highly oxygenated germacranolides, cernuumolides A–J, amomg which cernuumolide H (**302**) displayed the best cytotoxicity against HCT-116 cells with an IC_50_ value of 0.87 μM (Liu et al., 2016g).

**Figure 6:**
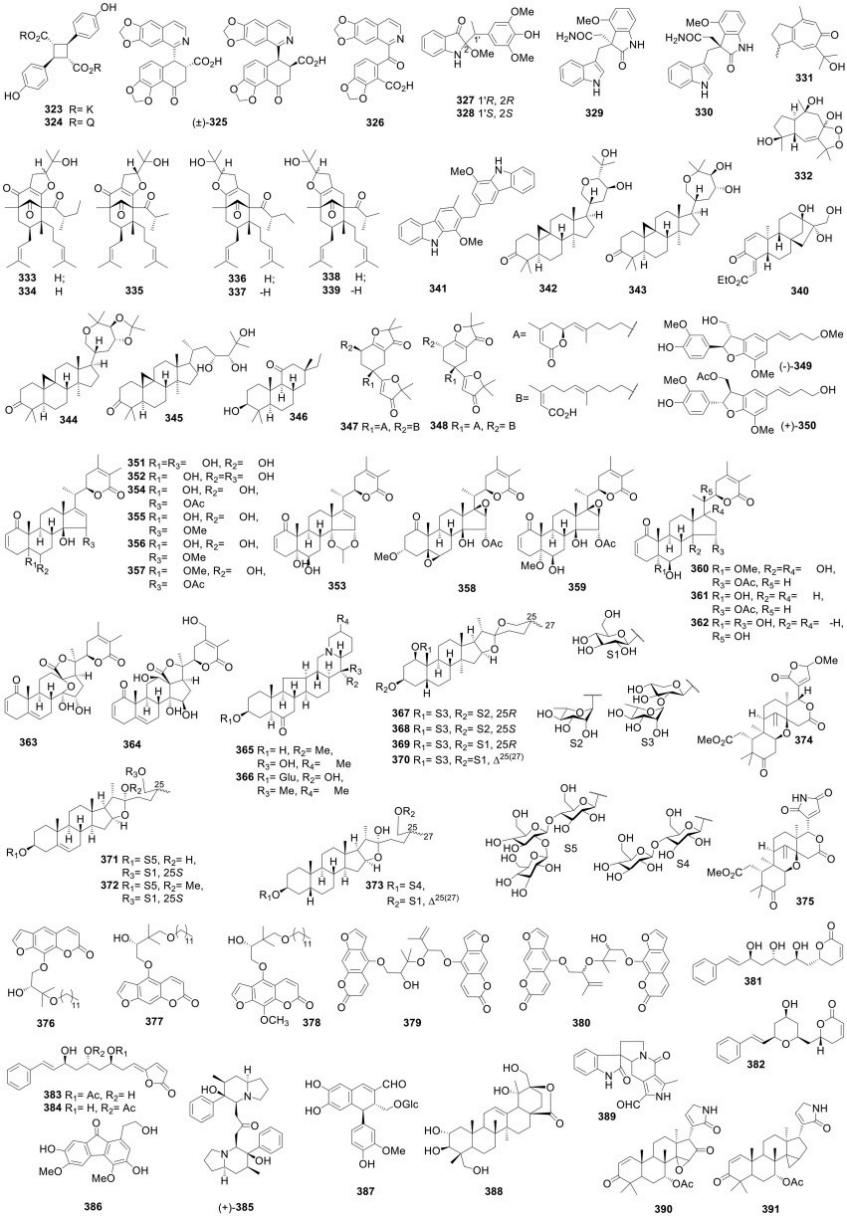
Structures of compounds **323-391**.

Sessilifols A–N, 14 new *ent*-abietane-type diterpenoids, together with three related new norditerpenoids, were obtained from *Chloranthus sessilifolius* (Fengqi Mountains, Sichuan, China). Sessilifols A (**303**) and B (**304**) possessed a rearranged skeleton. Sessilifol C (**305**) was a rare 7,8-seco-9-spiro-fused *ent*-abietane, while sessilifol O (**306**) represented the naturally-occurring 14-norabietane diterpenoid. Sessilifols F (**307**) and I (**308**) showed anti-neuroinflammatory activities against the NO production in LPS-stimulated murine BV-2 microglial cells with IC_50_ values of 8.3 and 7.4 μM (*N*-monomethyl-L-arginine (_L_-NMMA), 14.4 μM) (Wang et al., 2015h). Two heterodimers of an isopentenyl indole and a coumarin unit by a new fused cycloheptene linker, exotines A (**309**) and B (**310**), were obtained *Murraya exotica* roots. Compounds **309** and **310** inhibited the NO production in LPS-induced BV-2 microglial cells with IC_50_ values of 9.2 and 39.9 μM (positive control quercetin, 17.4 μM) (Liu et al., 2015a). One unusual sesterterpenoid (**311**) and four new sesquiterpene dimers (**312**-**315**) were afforded by *Inula britannica*. Compounds **311**-**315** showed inhibition on the NO production induced by LPS in RAW 264.7 macrophages with IC_50_ values of 10.86–49.44 μM (aminoguanidine, 7.90 μM) (Zhang et al., 2015l). Biginkgosides A–I (**316**-**324**) (Figure 6), new flavonol glycoside dimers possing a cyclobutane moiety, were isolated from the leaves of *Ginkgo biloba* (Chongming Island, China). Biginkgosides E (**320**) and H (**323**) showed inhibitory avtivities on NO production induced by LPS in BV-2 microglial cells with IC_50_ values of 2.91 and 17.23 μM (_L_-NMMA, 14.40 μM) (Ma et al., 2016a).

(±)-Hendersine A ((±)-**325**), a pair of novel isoquinoline alkaloids coupled by an isoquinoline and a succinic acid derivative, along with a new isoquinoline hendersine B (**326**), were isolated from *Corydalis hendersonii* (Tibet, China). (±)-Hendersine A ((±)-**325**) and hendersine B (**326**) exhibited a protective effect against the LPS-stimulated H9c2 myocyte injuries with 30–40% protection at 10 μM (quercetin, 45%) (Yin et al., 2016). An aqueous extract of *Isatis indigotica* leaves (Hebei, China) contained isatidifoliumindolinones A (**327**) and B (**328**), two enantiomers with the novel 2-[1′-(4″-hydroxy-3″,5″-dimethoxyphenyl)ethyl]-2-methoxyindolin-3-one carbon skeleton, which showed stereochemical-dependent inhibition on the LPS-induced NO production in BV2 cells with 1.2 and 21.4% inhibition at 10 μM (curcumin, 41.2%) (Li et al., 2016c). Interestingly, an aqueous extract of *Isatis indigotica* roots contained insatindibisindolamides A (**329**) and B (**330**), a pair of indole alkaloid enantiomers with a new bisindolylacetamide skeleton, which displayed antiviral activities against the Coxsackie virus B3 (CVB3) with the same IC_50_ value of 33.3 μM and selection index (SI) value of 5.3 (ribavirin, 292.5/6.8) (Liu et al., 2015g).

Rhizomes of *Curcuma phaeocaulis* (Chengdou, Sichuan, China) contained three new guaiane-type sesquiterpenes, phaeocaulisins K–M, along with phagermadiol. Phaeocaulisins L (**331**) and M (**332**) inhibited NO production induced by LPS in RAW 264.7 macrophages with IC_50_ values of 54.27 and 6.05 μM, respectively (hydrocortisone, 58.66 μM) (Ma et al., 2015b). Research into the flowers of *Hypericum monogynum* (Jiangsu, China) led to the identification of 10 rare methylated polycyclic polyprenylated acylphloroglucinol derivatives, hypermongones A–J. Hypermongones A–C (**333**–**335**) and hypermongones E-H (**336**-**339**) were found to be active against NO production induced by LPS in macrophages with IC_50_ values from 9.5 to 27.3 μM, better than that of _*L*_-NMMA (39.2 μM) (Xu et al., 2015c). Twigs of *Tricalysia fruticosa* (Xishuangbanna, Yunnan, China) afforded eight new cafestol-type diterpenoids, tricalysins A–H, among which tricalysin H (**340**) inhibited NO production in LPS-activated RAW 264.7 macrophages with an IC_50_ value of 6.6 μM (_L_-NMMA, 40.5 μM). Further investigation indicated tricalysin H (**340**) inhibited the expression of iNOS and production of the pro-inflammatory cytokines IL-6 and TNF-α through activation of NF-κB and phosphorylation of MAPKs (ERK, JNK and p38) (Shen et al., 2015). The leaves and stems of *Murraya tetramera* (Wuming, Guangxi, China) gave two unusual trimeric carbazole alkaloids, murratrines A and B, as well as 11 new carbazole dimers, murradines A–K. Murradine B (**341**) showed better inhibition on LPS-stimulated NO production in BV-2 microglial cells activity than quercetin (17.4 μM) with an IC_50_ of 11.4 μM (Lv et al., 2015). Three unique cyclolanostane triterpenoids **342**-**344** and six new isopimarane diterpenoids were isolated from the leaves and twigs of *Dysoxylum gotadhora* (Hainan, China). Compound **342** represented a naturally occurring 21,24-epoxy cyclolanostane-type triterpenoids, while compounds **343** and **344** were the first examples of 21,25-epoxy cyclolanostanetype triterpenoids. Triterpenoid **342** as well as isopimarane diterpenoids **345** and **346** were active against NO production induced by LPS in RAW 264.7 cells with IC_50_ values of 25.5, 27.4, and 14.5 μM (indomethacin, 21.5 μM) (Jiang et al., 2015a). Isolation of the root bark of *Aphanamixis grandifolia* (Yunnan, China) afforded 14 new diterpene dimers, aphanamenes C–P. Aphanamenes G (**347**) and I (**348**) exhibited potent inhibitory activities on NO production in RAW 264.7 macrophages with IC_50_ values of 7.75 and 8.86 μM (_L_-NMMA, 40.45 μM) (Zhang et al., 2015e).

A phytochemical study into the trunks of *Jatropha integerrima* (Guangdong, China) led to the isolation of two pairs of novel sesquineolignan enantiomers, (±)-jatrointelignans A and B, as well as one pair of new neolignan enantiomers, (±)-jatrointelignan D, and two new neolignans, (+)-jatrointelignan C and (+)-schisphenlignan I. (–)-Jatrointelignan D ((–)-**349**) and (+)-schisphenlignan I ((+)-**350**) inhibited the LPS-induced NO production in BV-2 microglial cells with IC_50_ values of 8.9 and 5.9 μM (quercetin,17.0 μM) (Zhu et al., 2015b). Sixteen new withanolides, physangulatins A–N, together with withaphysalins Y and Z were obtained from the leaves and stems of *Physalis angulata* (Guangxi, China). Physangulatins **351**-**362** as well as withaphysalins Y (**363**) and Z (**364**) were moderate active against the NO production with IC_50_ values of 3.51–71.69 μM (hydrocortisone, 58.79 μM) (Sun et al., 2016a). Research into the bulbs of *Fritillaria pallidiflora* (Xinjiang, China) gave four new isosteroidal alkaloids, yibeinones A–D. Yibeinones C (**365**) and D (**366**) showed potent inhibition on the Ach-induced contraction of rat isolated tracheas with EC_50_ values of 0.65 and 3.00 μM, better than nifedipine (6.50 μM) (Li et al., 2016j). Four new spirostanol saponins (Xiang et al., 2016a) and 10 new furostanol saponins (Xiang et al., 2016b) were identified from the rhizomes of *Tupistra chinensis* (Shennongjia, Hubei, China). Spirostanol saponins **367**-**370** and furostanol saponins **371**-**373** displayed inhibition on the LPS-stimulated NO production in RAW 264.7 macrophage cells with IC_50_ values of 3.1–46.2 μM (indomethacin, 47.4 μM). The stem bark of *Entandrophragma angolense* (Brong Ahafo Region, Ghana) afforded 16 new structurally-diverse limonoids, entangolensins A–P, among which entangolensins E (**374**) and K (**375**) inhibited the NO production induced by LPS in RAW 264.7 macrophages with IC_50_ values of 1.75 and 7.94 μM (_L_-NMMA, 32.55 μM) (Zhang et al., 2016h).

**Figure 7:**
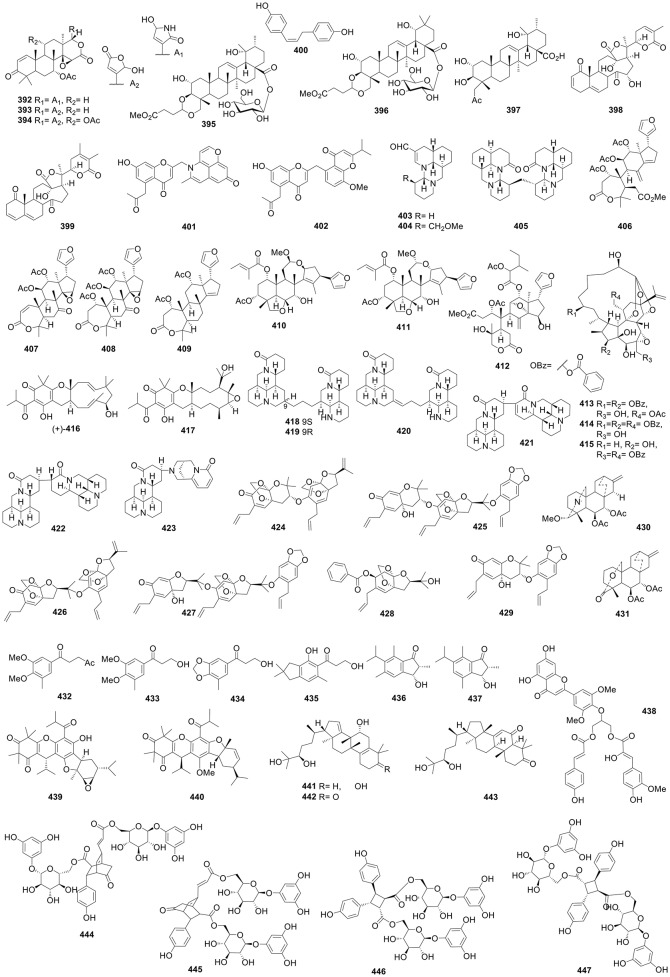
Structures of compounds **392-447**.

Two coumarins together with 10 coumarins with hydrophobic groups, andafocoumarins A–J, were afforded by *Angelica dahurica* cv. roots. (Hangbaizhi, Zhejiang, China). Andafocoumarins A–C (**376**–**378**) moderately inhibited NO production induced by LPS in mouse RAW 264.7 macrophage cells with IC_50_ values of 19.7, 13.9, and 25.9 μM, respectively (_L_-N_6_-(1-iminoethyl)-lysine, 23.7 μM) (Wei et al., 2016b). Three new dimeric furanocoumarins, dahuribiethrins H–J, were also isolated from roots of *Angelica dahurica* (Anhui, China). Dahuribiethrins H (**379**) and I (**380**) inhibited LPS-stimulated NO production in RAW 264.7 macrophage cells with IC_50_ values of 8.7 and 27.3 μM (indometacin, 38.6 μM) (Yang et al., 2017). Arylalkenyl α,β-unsaturated δ-lactone cryptoconcatones A–H, along with arylalkenyl α,β-unsaturated γ-lactone cryptoconcatones I and J, were isolated from the leaves and twigs of *Cryptocarya concinna* (Guangdong, China). Cryptoconcatones D (**381**), H (**382**), I (**383**), and J (**384**) moderately inhibited NO production in LPS-stimulated mouse RAW 264.7 macrophage cells with IC_50_ values of 3.2, 4.2, 3.4, and 7.5 μM respectively, better than L-NMMA (IC_50_ 45 μM) (Yang et al., 2016a). A pair of racemic indolizidine enantiomers, (±)-homocrepidine A, and a piperidine derivative, homocrepidine B, were obtained from stems of *Dendrobium crepidatum* (Yunnan, China). (+)-Homocrepidine A ((+)-**385**) showed inhibition on the LPS-induced NO production in RAW 264.7 macrophages with an IC_50_ value of 3.6 μM (indomethacin, IC_50_ 42.2 μM) (Hu et al., 2016c). 1-Ethoxy-3,7-dihydroxy-4,6-dimethoxy-9-fluorenone (**386**) was obtained from the roots of *Litsea cubeba* (Anhui, China), and showed a TNF-α inhibition with an IC_50_ value of 28.2 μM (Lin et al., 2016a). Vitexnegheteroins E–G, three new phenylnaphthalene-type lignans, and vitexnegheteroin H, a new polyoxygenated ursane-type triterpene, were identified from the seeds of *Vitex negundo* var. *heterophylla* (Huludao, Liaoning, China). Vitexnegheteroins E (**387**) and H (**388**) displayed activities against LPS-induced NO production with IC_50_ values of 17.27 and 17.23 μM (positive control SMT, 1.83 μM) (Hu et al., 2016b). The stems of *Nauclea officinalis* (Hainan, China) contained two novel indole alkaloids, namely nauclealises A and B. Nauclealise A (**389**) showed activity against the LPS-induced NO production in RAW 264.7 macrophages with an IC_50_ value of 0.82 μM, more active than aminoguanidine (1.80 μM) (Chen et al., 2016a). Investigation into the root bark of *Toona sinensis* (Sichuan, China) led to the isolation of 12 new azadirone-type and gedunin-type limonoids, toonasinemines A–K. Toonasinemines A (**390**), B (**391**), F (**392**), H (**393**), and I (**394**) (Figure 7) displayed inhibitory activity against the LPS-induced NO production in RAW 264.7 macrophages with IC_50_ values of 10.21–20.68 μM (_L_-NMMA, 32.55 μM) (Li et al., 2016e).

Three new pentacyclic triterpenes **395**-**397** were discovered from the acorns of *Quercus serrata* var. *brevipetiolata* (Dabie Mountains, Anhui, China) and showed activity against the LPS-induced NO production in RAW 264.7 macrophages with IC_50_ values of 8.2, 12.8, and 19.1 μM, respectively, more effective than indometacin (47.4 μM) (Huang et al., 2016). 13,14-*Seco*-withanolide minisecolides A–D were identified from the whole plants of *Physalis minima* (Anhui Province, China). Minisecolides B (**398**) and C (**399**) inhibited LPS-induced NO production in RAW 264.7 macrophages with IC_50_ values of 25.34 and 20.81 μM, respectively (_L_-NMMA, 30.11 μM) (Lin et al., 2016c). One new diphenol and two new isocoumarin carbonates were obtained from the aerial parts of *Lawsonia inermis* (Taiwan, China), among which compound **400** exhibited inhibition on the LPS-induced NO production in RAW 264.7 cells with an IC_50_ value of 5.63 μg/mL, more effective than indomethacin (IC_50_, 78.56 μg/mL) (Yang et al., 2016b). Two novel bischromones, fistulains A (**401**) and B (**402**), were isolated from the bark of *Cassia fistula*. Fistulain A (**401**) derived from a chromone and a tricyclic alkaloid through a unique C-14–N linkage, while istulain B (**402**) had an unusual C-14–C-5′ linkage. Fistulain A (**401**) displayed anti-TMV (tobacco mosaic virus) activity with an IC_50_ value of 43.8 μM (ningnamycin, 52.4 μM) (Zhou et al., 2015c).

Myrifamines A–C (**403**-**405**), three new myrioneuron alkaloids with unusual carbon skeletons, were obtained from *Myrioneuron faberi* (Sichuan, China). Myrifamine C (**405**) was the first example of symmetric dimers of the myrioneuron alkaloids (Cao et al., 2015b). Research into the whole plants of *Munronia henryi* (Wenshan, Yunnan, China) afforded 14 new limonoids, munronins A–N. Munronins B (**406**) and H–L (**407**-**411**) showed anti-TMV activities with IC_50_ values ranging from 19.6 to 44.4 μg/mL (ningnanmycin, 44.6μg/mL). Munronin A (**412**) exhibited cytotoxic effects on HL-60 and SW480 cells with IC_50_ values of 0.44 and 0.86 μM, respectively (Yan et al., 2015b). Seven novel daphnane diterpenoids, stelleralides D–J, were obtained from the roots of *Stellera chamaejasme* (Baotou, Inner Mongolia, China). Stelleralides F (**413**), G (**414**), and H (**415**) exhibited anti-HIV activities with EC_50_ values of 0.93, 0.73, and 0.98 nM and SI values of >10,000, much more potent than zidovudine (EC_50_ 32 nM, SI ≥ 116) (Yan et al., 2015a). Seven filicinic acid-based meroterpenoids comprised 6/6/11, 6/6/7/5, or 6/6/10 ring systems were obtained from *Hypericum japonicum*. (+)-Hyperjaponols B ((+)-**416**) and D (**417**) exhibited anti-Epstein-Barr virus activities with EC_50_ values of 0.57 and 0.49 μM, respectively, better than ganciclovir (IC_50_, 2.86 μM) (Hu et al., 2016a).

Flavesines A–F (**418**-**423**), six unusual matrine-type alkaloid dimers, were obtained from the roots of *Sophora flavescens* (Shaanxi, China) and displayed inhibitoriy activities against hepatitis B virus with IC_50_ values of 17.16–86.60 μM (foscarnet, 105 μM) (Zhang et al., 2016l). The roots of the *Illicium oligandrum* (Guangxi, China) contained 10 novel prenylated C_6_-C_3_ compounds, namely illioliganpyranones B–G, illioliganones J–K, illioliganpyranol A, and illioliganfuranol A. Illioliganpyranones B (**424**), C (**425**), E (**426**), and F (**427**) as well as illioliganfuranol A (**428**) showed significant activities against CVB3 virus with IC_50_ and SI value ranges of 3.70–11.11 μM and 5.2–21.1, respectively (ribavirin, 2.12 mM/3.87). Illioliganpyranone D (**429**) exhibited potent activity against H3N2 influenza virus A with an IC_50_ value of 5.55 μM and SI value of 18.0 (oseltamivir, 4.39 μM/700) (Ma et al., 2016c). Whole plants of *Spiraea japonica* var. *acuminata* (Yunnan, China) contained five new diterpenes, among which spirimine B (**430**) and spiramilactone F (**431**) showed anti-TMV virus activities with protective/curative rates of 92.91%/41.30% and < 20%/69.40% at 100 μg/mL, respectively (ningnanmycin at 48.20%/50.72%) (Ma et al., 2016f). Research into whole plants of *Lavandula angustifolia* (Yunnan, China) afforded three novel phenylpropanoids **432**-**434** with 33.9–38.4% inhibition on the TMV infection at 20 μM (ningnanmycin, 33.6%) (Tang et al., 2016a).

Tabasesquiterpene B (**435**) (Shang et al., 2016), nicosesquiterpenes A (**436**) and B (**437**) (Shen et al., 2016) were obtained from leaves of *Nicotiana tabacum* (Yunnan, China), and showed anti-TMV activities with 35.2, 36.7, and 45.6% inhibition at 20 μM, respectively, better than ningnanmycin. Ananasin A (**438**), a flavonolignan, was discovered from *Ananas comosus* (Hainan, China), and was active against *S. aureus* and *E. coli* with the same MIC value of 0.156 μg/mL (ciprofloxacin, MIC 0.156 μg/mL) (Huang et al., 2015). Callistrilones A (**439**) and B (**440**) with an unprecedented [1]benzofuro-[2,3-a] xanthene or [1]benzofuro [3,2-b]xanthene pentacyclic ring system was obtained from the leaves of *Callistemon rigidus* (Guangdong, China). Callistrilone A (**439**) showed antibacterial activity against multiresistant *S. aureus* ATCC33591, *S. aureus* Mu50, and *Enterococcus faecium* 13-01 with IC_50_ values of 16–32 μg/mL, more potent than oxacillin (IC_50_, 256–512 μg/mL) (Cao et al., 2016b). Tetracyclic triterpenoids, ricinodols A–G, were isolated from the stems and leaves of *Ricinodendron heudelotii* (Hainan, China). Ricinodols A (**441**) and B (**442**) possessed a novel concurrent rearrangement of Me-19 (10→9) and Me-30 (14→8). Ricinodol E (**443**) inhibited both human and mouse 11β-hydroxysteroid dehydrogenase type 1 (11β-HSD1) with IC_50_ values of 0.36 and 0.84 μM, respectively (Yu et al., 2015).

**Figure 8:**
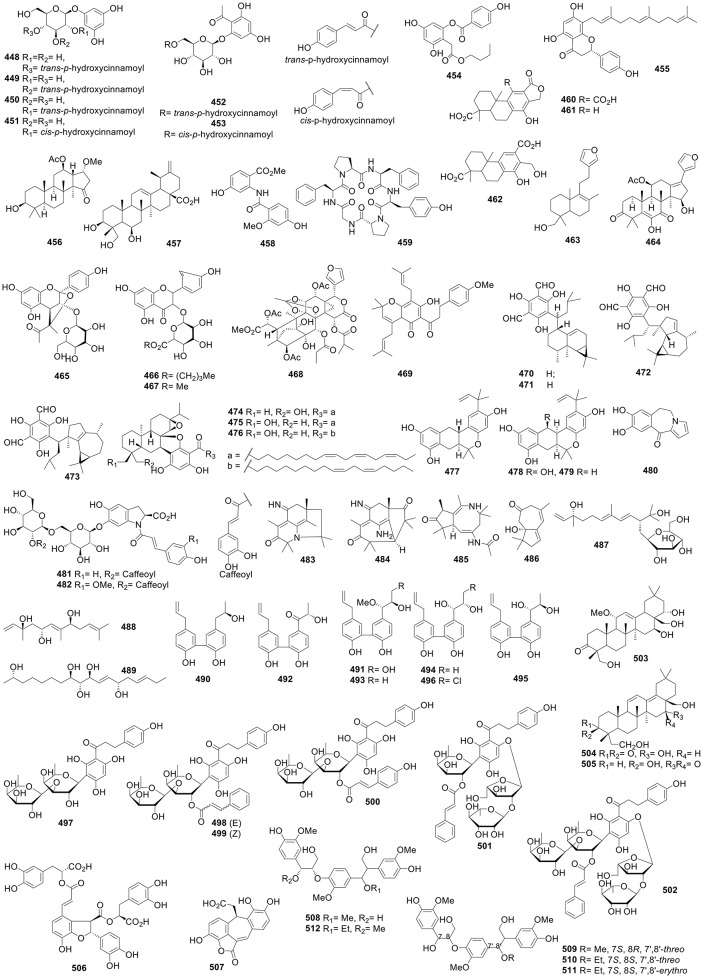
Structures of compounds **448-512**.

Ten new phenylpropanoid glucosides, tadehaginosides A–J (**444**-**453**) (Figure 8), were obtained from *Tadehagi triquetrum* (Hainan, China). Tadehaginosides A (**444**) and B (**445**) had an unusual bicyclo[2.2.2]octene skeleton, while tadehaginosides C (**446**) and D (**447**) contained a unique cyclobutane basic core in their carbon scaffolds. Tadehaginosides C–J (**446**-**453**), particularly tadehaginoside D (**447**), significantly increased the basal and insulin-elicited glucose uptake, with an efficacy comparable to 100 nM of insulin (Zhang et al., 2016i). New depside derivatives were obtained from the flowers of *Impatiens balsamina* (Nanjing, Jiangsu, China), among which compound **454** showed α-glucosidase inhibition with an IC_50_ value of 0.72 μg/mL (acarbose, 3.36 μg/mL) (Li et al., 2015e). The root bark of *Morus alba* var. *tatarica* (Xinjiang, China) afforded four new flavonoids, mortatarins A–D, among which mortatarin D (**455**) showed potent α-glucosidase inhibition with an IC_50_ value of 5.0 μM (genistein, 17.8 μM) (Zhang et al., 2015m).

Hupehenols A–E, new 20,21,22,23,24,25,26,27-octanordam marane triterpenoids, were isolated from *Viburnum latifolia* (Caojian, Yunnan, China). Interestingly, all the triterpenoids selectively inhibited human 11β-HSD1 with IC_50_ values of 15.3–194.5 nM, while the IC_50_ range for 11β-HSD2 was 11.3–299.6 μM. The SI (the IC_50_ ratio of HSD2/HSD1) for hupehenols A–E ranged from 131.0 to 3,967.0, much higher than that of glycyrrhetinic acid (GA, 0.1), indicating these compounds were highly selective inhibitors of human 11β-HSD1. Hupehenol B (**456**) even showed better activity against human 11β-HSD1 with an IC_50_ of 15.3 nM than GA (29.1 nM) (Chen et al., 2015b). A new ursane triterpene acid (**457**), together with a new oleanane triterpene acid, was isolated from the whole plants of *Spermacoce latifolia* (South China Botanical Garden, Guangdong, China). Compound **457** showed α-glucosidase inhibition with an IC_50_ value of 0.42 mM, equivalent to acarbose (0.409 mM) (Luo et al., 2015c). Research into the seeds of *Vaccaria hispanica* (Anguo, Hebei, China) led to the discovery of new diantheramide (**458**) and segelin I (**459**), both which showed significant α-glucosidase inhibition with IC_50_ values of 0.08 and 0.28 mM, respectively (acarbose, 0.41 mM) (Zheng et al., 2015a). Roots of *Phlomis tuberose* (Shangdu Town, Inner Mongolia, China) afforded new diterpenoids **460**-**463** that exhibited better α-glucosidase inhibitory activities than acarbose (3.72 mM) with IC_50_ of 0.067–0.379 mM (Yang et al., 2015d). The twigs of *Dysoxylum mollissimum* (Ledong, Hainan, China) contained new limonoids, dysoxylumosins A–M. Dysoxylumosin F (**464**) displayed an human 11β-HSD1 with an IC_50_ value of 9.6 nM (glycyrrhetinic acid, 8.8 nM) (Zhou et al., 2015a).

Flavone derivatives, falandiosides A (**465**) and B (**466**), and glucuronide (**467**), were isolated from the fruits of strawberry (Guangdong, China). Compounds **465**–**467** showed modest antioxidation against ABTS radical with IC_50_ values of 5.74, 8.80, and 4.42 μM, respectively (_*L*_-ascorbic acid, 14.21 μM). Compounds **466** and **467** also showed α-glucosidase inhibition with IC_50_ values of 107.52 and 65.22 μM, respectively (acarbose, 619.94 μM) (Yang et al., 2016d). Two new phragmalin-type limonoids were obtained from the stems of *Chukrasia tabularis* A (Hainan, China). Compound **468** showed α-glucosidase inhibitory activity with an IC_50_ value of 0.96 mM (acarbose, 0.95 mM) (Peng et al., 2016a). Philippin C (**469**) was from the root bark of *Flemingia philippinensis* and showed potent inhibitory activity of protein tyrosine phosphatase 1B (PTP1B) with an IC_50_ value of 6.5 μM (ersolic acid, 15.5 μM) (Wang et al., 2016g). New conjugates of sesquiterpenoids and acylphloroglucinols were isolated from the leaves of *Eucalyptus robusta* (Guangxi, China), among which eucarobustols A (**470**) and B (**471**) were the first examples of conjugates with aristolane and acylphloroglucinol units. Eucarobustols A (**470**), C (**472**), and D (**473**) displayed PTP1B inhibition with IC_50_ values of 1.3, 1.8, and 1.6 μM (oleanolic acid, 2.3 μM), respectively (Yu et al., 2016c).

Roots of the rare chloranthaceae plant *Chloranthus oldhamii* (Jinggang Mountains, Jiangxi, China) contained chlorabietols A–C (**474**-**476**), three abietane-type diterpenoids linked with different alkenyl phloroglucinol units through forming a 2,3-dihydrofuran ring. Compounds **474**-**476** showed PTP1B inhibition with IC_50_ values of 12.6, 5.3, and 4.9 μM, respectively (oleanolic acid, 3.2 μM) (Xiong et al., 2015a). The stems of *Artocarpus nanchuanensis* (Jinfoshan Mountain, Chongqing, China) contained four new stilbene derivatives, hypargystilbenes B–E, among which hypargystilbenes B (**477**), D (**478**), and E (**479**) were inhibitory toward PTP1B with IC_50_ values of 3.23, 37.31, and 2.53 nM, respectively (oleanolic acid, 1.60 nM) (Zhang et al., 2015j). Portulacatone (**480**) (Yue et al., 2015), oleraceins K (**481**) and L (**482**) (Jiao et al., 2015b), isolated from *Portulaca oleracea* (Shandong, China) displayed dose-dependent DPPH radical scavenging activities with EC_50_ values of 14.36, 15.30, and 16.13 μM, better than vitamin C. *Portulaca oleracea* was also the source of the new alkaloids, oleracimine (**483**), oleracimine A (**484**), and oleracone A (**485**), as well as azulene compound, oleracone B (**486**) (Li et al., 2016b).

**Figure 9:**
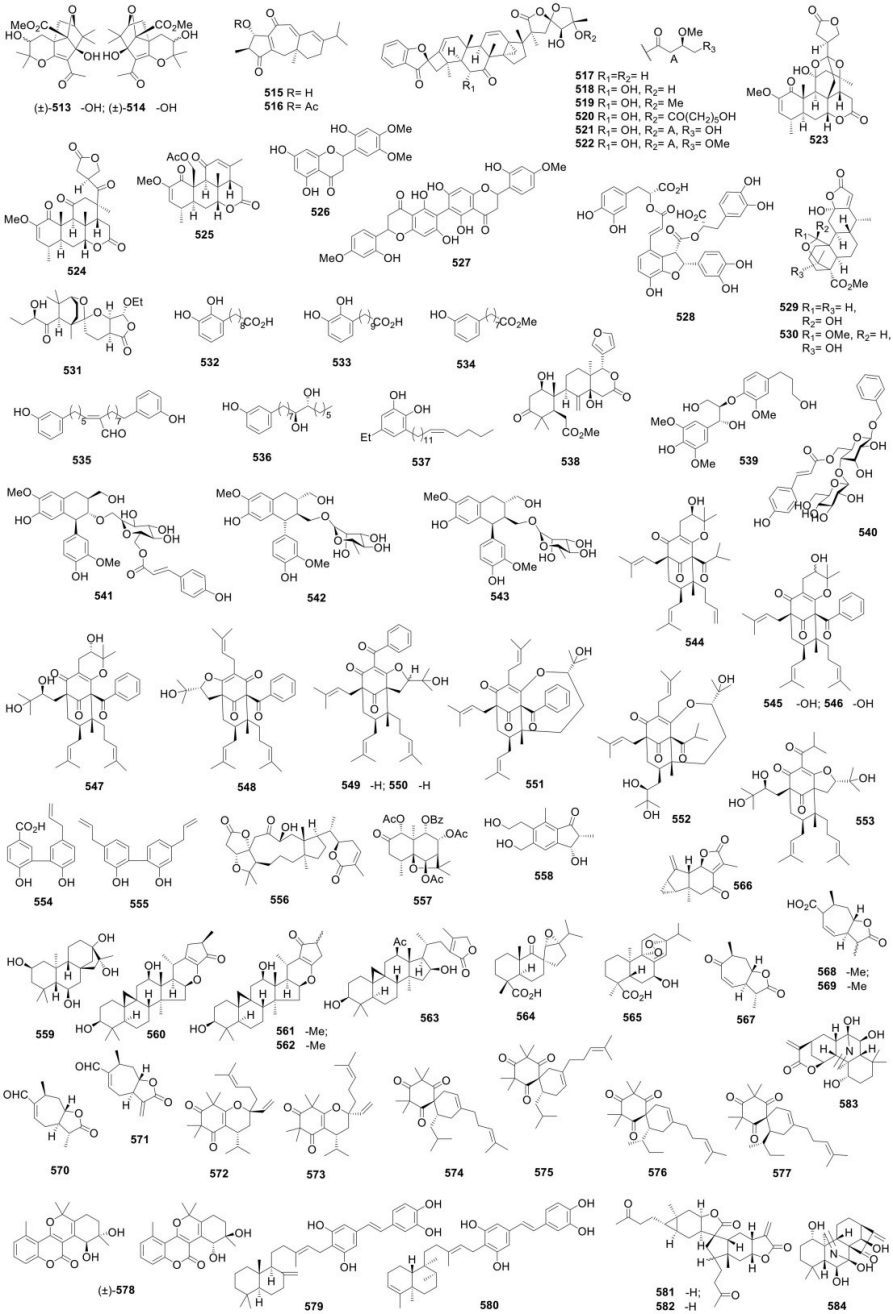
Structures of compounds **513-584**.

*Murraya koenigii* (Xishuangbanna, Yunnan, China) afforded four new alkenes, three of which (**487**-**489**) showed antioxidative activities against DPPH radical with IC_50_ values of 38.4, 23.5, and 25.4 μM, respectively (chlorogenic acid, 56.4 μM) (Ma et al., 2016b). Clypearianins A–G (**490**-**496**), seven new 3,3′-neolignans, were discovered from the twigs and leaves of *Portulaca clypearia* (Guangxi, China), and exhibited significant ABTS radical scavenging activity with IC_50_ values ranging from 4.3 to 14.9 μg/mL (trolox, 14.1 μg/mL) (Lou et al., 2016). Six new dihydrochalcone C-glycosides, carambolasides E–J (**497**–**502**), were obtained from the fruits of *Averrhoa carambola* (Guangdong, China) and displayed potent ABTS radical scavenging activity with IC_50_ values ranging from 2.54 to 4.52 μM, more effective than _L_-ascorbic acid (14.21 μM) (Yang et al., 2016c). Research into the roots of *Bupleurum chinense* afforded 17 triterpenoids, three of which oleanane triterpenes (**503**–**505**) exhibited neuroprotective effects against H_2_O_2_-induced SH-SY5Y cell death (Li et al., 2016d). *Salvia miltiorrhiza* afforded three new minor phenolic acids and six known compounds, and new compound **506** showed 84.3% scavenging rate of DPPH radical at 2 mM, better than vitamin C (74.9%) (Si et al., 2016). Two new norditerpenoids, miltiolactones A and B, and seven new neolignans, miltiolignanolides A–G, were obtained from the root of *Salvia miltiorrhiza* (Shandong, China). Miltiolignanolide C (**507**) displayed ABTS radical scavenging activity with an IC_50_ value of 3.73 μM (trolox, 5.10 μM) (Li et al., 2016g). Seven new neolignans were isolated from the seeds of *hawthorn*, among which compounds **508**-**512** acted as modest ABTS-radical scavengers with IC_50_ values ranging from 4.4 to 7.9 μM (trolox, 18.2 μM) (Peng et al., 2016c). (±)-Melicolone A ((±)-**513**) (Figure 9) and (±)-melicolone B ((±)-**514**), a pair of rearranged prenylated acetophenone epimers with an unprecedented 9-oxatricyclo-[3.2.1.1^3, 8^]nonane core, were afforded by *Melicope ptelefolia* leaves (Southeast Asia) and displayed cell protecting activities against oxidative stress in human vein endothelial cells induced by high glucose, equivalent to resveratrol at 5 μM (Xu et al., 2014). Jatrocurcadiones A (**515**) and B (**516**) possessing a novel 10,11-seco-premyrsinane diterpenoid skeleton, were obtained from the twigs of *Jatropha curcas*. Jatrocurcadione A (**515**) showed inhibition of thioredoxin reductase (TrxR) with an IC_50_ of 10.0 μM (curcumin, 25.0 μM) (Bao et al., 2015).

Phainnanoids A–F (**517**-**522**) incorporating 4,5- and 5,5-spirocyclic unique motifs, were isolated from *Phyllanthu hainanensis* (Hainan, China), among which phainanoid F (**522**) showed the best immunosuppressive activities *in vitro* against the proliferation of T and B lymphocytes with IC_50_ values of 2.04 and 1.60 nM, respectively (CsA, IC_50_ 14.21 and 352.87 nM) (Fan et al., 2015b). A new 20*S*- quassinoid with an unusual cagelike 2,4-dioxaadamantane nucleus and a migrated side chain, perforalactone A (**523**), together with perforalactones B (**524**) and C (**525**), were produced by twigs and stems of *Harrisonia perforata*. Compounds **523** and **524** showed inhibitory activites against nicotinic acetylcholine receptor (nAChR) with IC_50_ values of 15.8 and 1.26 nM, respectively (imidacloprid, 0.79 nM) (Fang et al., 2015).

The roots of *Artocarpus heterophyllus* (Nanning, Guangxi, China) contained two new flavanones, artoheterones A (**526**) and B (**527**), which inhibited the respiratory burst of rat PMNs with IC_50_ values of 1.67 and 0.19 μM, respectively (quercetin, 4.68 μM) (Ren et al., 2015a). Salvianolic acid Y (**528**) from the dried roots of *Salvia officinalis* protected PC12 cells from H_2_O_2_-induced injury with 54.2% protection rate at 10 μM (salvianolic acid B, 35.2%) (Gong et al., 2015). Caesalsappanins A–L, 12 new cassane-type diterpenes, were isolated from the seeds of *Caesalpinia sappan* (Nanning, Guangxi, China), among which caesalsappanins G (**529**) and H (**530**) exhibited antimalarial activities against the chloroquine-resistant *P. falciparum* K1 with IC_50_/SI values of 0.78/17.6 and 0.52/16.4 μM, respectively (Chloroquine, IC_50_/SI 0.37/129.5 μM) (Ma et al., 2015a). Leonuketal (**531**) possessing an unprecedented tetracyclic diterpenoid skeleton within a spiroketal moiety, was isolated from the aerial parts of *Leonurus japonicas* and displayed significant vasorelaxant activity against contraction of rat aorta induced by KCl with an EC_50_ value of 2.32 μM (methoxyverapamil, 0.58 μM) (Xiong et al., 2015b).

Eight new urushiol-type compounds were isolated from the resins of *Toxicodendron vernicifluum* (Bozhou, Anhui, China), among which compounds **532**-**537** inhibited AA-induced platelet aggregation with IC_50_ values of 3.09–11.83 μM (aspirin, 25.59 μM) (Xie et al., 2016a). Research into the seeds of *Khaya senegalensis* (Guangdonge, China) afforded 12 new limonoids, khasenegasins O–Z. Khasenegasin Z (**538**) exhibited protection for the injury induced by glutamate in primary rat cerebellar granule neuronal cells by increasing 83.3% and 80.3% viability at 10 μM and 1 μM, respectively (edaravone, 86.7% at 50 μM) (Tian et al., 2016c). Three new phenolic compounds were obtained from the roots of *Alangium chinense* (Guangxi, China). Compound **539** showed moderate inhibition against rat liver microsomal lipid peroxidation induced by Fe^2+^-cysteine with an IC_50_ value of 8.18 μM (vitamin E, 54.2 μM) (Zhang et al., 2016k). Compounds **540**-**543** were isolated from the aerial parts of *Lespedeza cuneata* (Henan, China) and active toward the transcription of XBP1 with EC_50_ values from 0.18 to 0.64 μM (Zhou et al., 2016b). Eleven new PPAPs, uraliones A–K, were afforded by whole plants of *Hypericum uralum* (Yunnan, China). Almost all compounds (**544**-**553**) were active against corticosterone-induced PC12 cell injury except uralione I (Zhou et al., 2016e). Tzumins A (**554**) and B (**555**), two novel lignan derivatives, were obtained from the bark of *Sassafras tzumu* (Guangxi, China), and displayed potent AChE inhibition with IC_50_ values of 2.00 and 1.81 μM, respectively (galanthamine, 2.99 μM) (Lu et al., 2017).

Stems of *Schisandra pubescens* (Jinfo mountain, Chongqing, China) contained a new triterpenoid **556** with hepatoprotective activity against D-GalN-induced cell injury in QSG7701 cells with 60.5% survival rates at 10 μM (silybin, 66.2%) (Wang et al., 2016a). The seeds of *Celastrus monospermus* (Guangdong, China) afforded 15 novel β-dihydroagarofuran-type sesquiterpenes, among which celaspermin E (**557**) showed lifespan extending effects of *C. elegans* with an extention rate of 37% at 50 μM, similar to the positive control rapamycin (38%) (Gao et al., 2016a). The aerial parts of *Pteris cretica* (Guizhou, China) afforded four new pterosin sesquiterpenoids and a new *ent*-kaurane diterpenoid. Compounds **558** and **559** showed more potent lipid-lowering activity than the positive control berberine in 3T3-L1 adipocyte (Luo et al., 2016b). Cimyunnins A–D (**560**-**563**) and cimyunnin D (**563**), characterized a fused cyclopentenone ring G and a rearranged γ-lactone ring F, respectively, were identified from the fruit of *Cimicifuga yunnanensis* (Daocheng, Sichuan, China). Cimyunnin A (**560**) displayed a similar anti-angiogenic activity as sunitinib both *in vitro* and *ex vivo* (Nian et al., 2015). Macrophypenes A–E, five new diterpenoids, were isolated from leaves of *Callicarpa macrophylla* (Guangxi, China). Macrophypene A (**564**) was a novel spiroditerpenoid, while macrophypene E (**565**) was a rare *ent*-abietane diterpenoid with a peroxide bridge (Xu et al., 2015a).

Eight new sesquiterpenes and two new lignans, were isolated from the fruits of *Xanthium sibiricum* (Helen City, Heilongjiang, China). Sibirolide A (**566**) was the first example of a 3/5/6/5 tetracyclic eudesmane sesquiterpene lactone formed at C-6 and C-7, and norxanthantolide B (**567**) was the first example of the naturally-occuring xanthane tetranorsesquiterpene, while norxanthantolides C–F (**568**-**571**) were the first xanthane trinorsesquiterpenes to date (Shi et al., 2015). Six novel Diels-Alder adducts of a polymethylated phloroglucinol derivative with myrcene, calliviminones C–H (**572**–**577**), were produced by fruits of *Callistemon viminalis* (Guangdong, China) (Wu et al., 2015f). A pair of coumarin enantiomers ((±)-**578**) with a rare polycyclic pyrano[3-2c] skeleton were isolated from the whole plants of *Ainsliaea fragrans* (Shiyany, Hubei, China) (Xue et al., 2015). Denticulatains A (**579**) and B (**580**), two novel heterodimers formed from a diterpene and a stilbene, were isolated from *Macaranga denticulate* (Yang et al., 2015b). Dicarabrones A (**581**) and B (**582**), a pair of epimers of two sesquiterpene lactone units linked by a cyclopentane ring, were isolated from the whole plants of *Carpesium abrotanoides* (Wu et al., 2015b).

**Figure 10:**
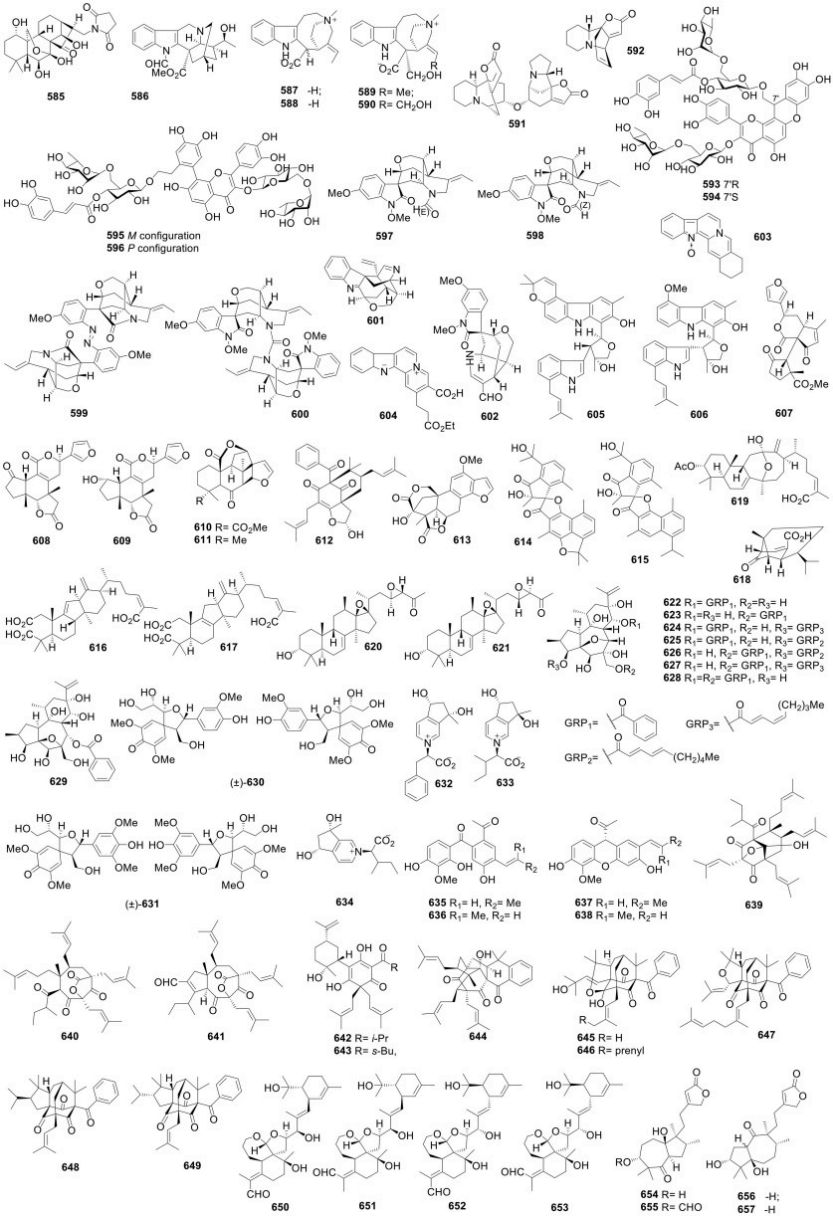
Structures of compounds **585-657**.

Three new diterpene alkaloids, kaurines A–C (**583**-**585**) (Figure 10), were obtained from *Isodon rubescens* (Jianshi County, Hubei, China). Kaurines A (**583**) and B (**584**) had a unique 7,20-aza-*ent*- kaurane skeleton, while kaurine C (**585**) contained a rare succinimide moiety (Liu et al., 2015f). Monoterpenoid indole alkaloid ervatamines A–I, were isolated from *Ervatamia hainanensis* (Tunchang, Hainan, China). Ervatamine A (**586**) was a ring-C-contracted ibogan-type monoterpenoid indole alkaloid with an unusual 6/5/6/6/6 pentacyclic system. Ervatamines B–E (**587**-**590**) displayed a rare aza- 9/6 ring system (Zhang et al., 2015c). Flueggether A (**591**) and virosinine A (**592**) were obtained from a Chinese medicinal plant *Flueggea virosa*. Flueggether A (**591**) represented the first example of a securinega alkaloid oligomer with an ether bridge and virosinine A (**592**) had a new heterocyclic backbone (Zhang et al., 2015f). Forsythoneosides A–D (**593**-**596**), four unusual adducts of a flavonoid unit fused to a phenylethanoid glycoside through a pyran ring or carbon-carbon bond, together with four new phenylethanoid glycosides, were isolated from the fruits of *Forsythia suspense* (Yuncheng, Shanxi, China) (Zhang et al., 2015d). The first rotameric monoterpenoid indole alkaloids (MIAs) **597** and **598**, along with two dimeric MIAs **599** and **600** linked by a azo- and an urea unit, respectively, were isolated from roots of *Gelsemium elegans* (Conghua, Guangdong, China) (Zhang et al., 2015k). Leaves and vine stems of *Gelsemium elegans* (Xishuangbanna, Yunnan, China) afforded nine new koumine-, humantenine-, and yohimbine- type alkaloids as well as 12 known analogs. Compound **601** was the first example of *N*-4-demethyl koumine type alkaloid, orhumantenine A (**602**) was the first norhumantenine alkaloid, and compounds **603** and **604** were the first *N*-1-oxide and seco-E-ring yohimbane type alkaloids, respectively (Xu et al., 2015d).

Stems of *Glycosmis pentaphylla* afforded glycosmisines A (**605**) and B (**606**), two unique alkaloids dimeric from a carbazole unit and a indole unit (Chen et al., 2015c). Three rearranged labdane-type diterpenoids, hapmnioides A–C (**607**–**609**), were obtained from *Haplomitrium mnioides* (Guizhou, China) (Zhou et al., 2016c), Two unprecedented labdane-type diterpenoids with a six-rings' system, haplomintrins A (**610**) and B (**611**), were also obtained from *Haplomitrium mnioides* (Zhou et al., 2015b). Nine new PPAPs, hyperattenins A–I, were obtained from the aerial parts of *Hypericum attenuatum* (Qichun, Hubei, China). Hyperattenin A (**612**) was characterized as a bicycle bicyclo[3.3.1]nonane derivative containing a rare hemiacetal functionality (Li et al., 2015a). A new rearranged 17-norpimarane, icacintrichantholide **613**, was isolated from *Icacina trichantha* (Zhao et al., 2015a). Three dimeric cadinane sesquiterpenoids, involucratustones A–C, were produced by the rhizomes of *Stahlianthus involucratus*. Involucratustones A (**614**) and B (**615**) were two rearranged homodimers fused with a unique 1-oxaspiro[4.4]nonane core, and involucratustone C was a novel 3′,4′-seco-cadinane-dimer (Li et al., 2015d). Ten new triterpene acids, kadcoccinic acids A–J, were afforded by stems of *Kadsura coccinea* (Ziyuan, Guangxi, China). Kadcoccinic acids A (**616**) and B (**617**) were the first examples of 2,3-seco-6/6/5/6-fused tetracyclic triterpenoids (Liang et al., 2015).

The stems of *Kadsura coccinea* afforded kadcoccinin A (**618**), a sesquiterpenoid within a tricyclo[4.4.0.0^3, 10^]decane scaffold (Hu et al., 2016d). *Kadsura coccinea* was also the source of six new lanostane-related triterpenoids, kadcoccinones A–F. Kadcoccinone C (**619**) possessed an unprecedented 6/6/9-fused carbocyclic core containing a rare oxabicyclo[4.3.1]decane system, while kadcoccinones D (**620**) and E (**621**) were two novel 18(13→12)-abeo-26-norlanostane triterpenoids (Hu et al., 2015). Neogenkwanines A–H (**622**-**629**), daphnane-type diterpenes with a 4,7- or 4,6-oxo bridge, were isolated from *Daphne genkwa* (Mianyang, Sichuan, China) (Li et al., 2015c). (±)-Subaveniumins A ((±)-**630**) and B ((±)-**631**), two pairs of racemic neolignans with a rare 2-oxaspiro[4.5] deca-6,9-dien-8-one motif, were isolated from the bark of *Cinnamomum subavenium* (Laifeng, Hubei, China) (Lai et al., 2015). Three new zwitterionic alkaloids, ningpoensines A–C (**632-634**), were obtained from the roots of *Scrophularia ningpoensis* (Zhang et al., 2015g). Two pairs of rearranged *cis*/*trans* neolignane isomers, penchinones A–D (635–638), were identified from *Penthorum chinense* (Guling, Sichuan, China), among which penchinones C (**637**) and D (**638**) featured an unprecedented 7,30-neolignane carbon skeleton (He et al., 2015). Eleven new PPAP-type derivatives, hyphenrones G–Q (**639**-**649**), were obtained from *Hypericum henryi* (Dongchuan, Yunnan, China) (Yang et al., 2015c).

**Figure 11:**
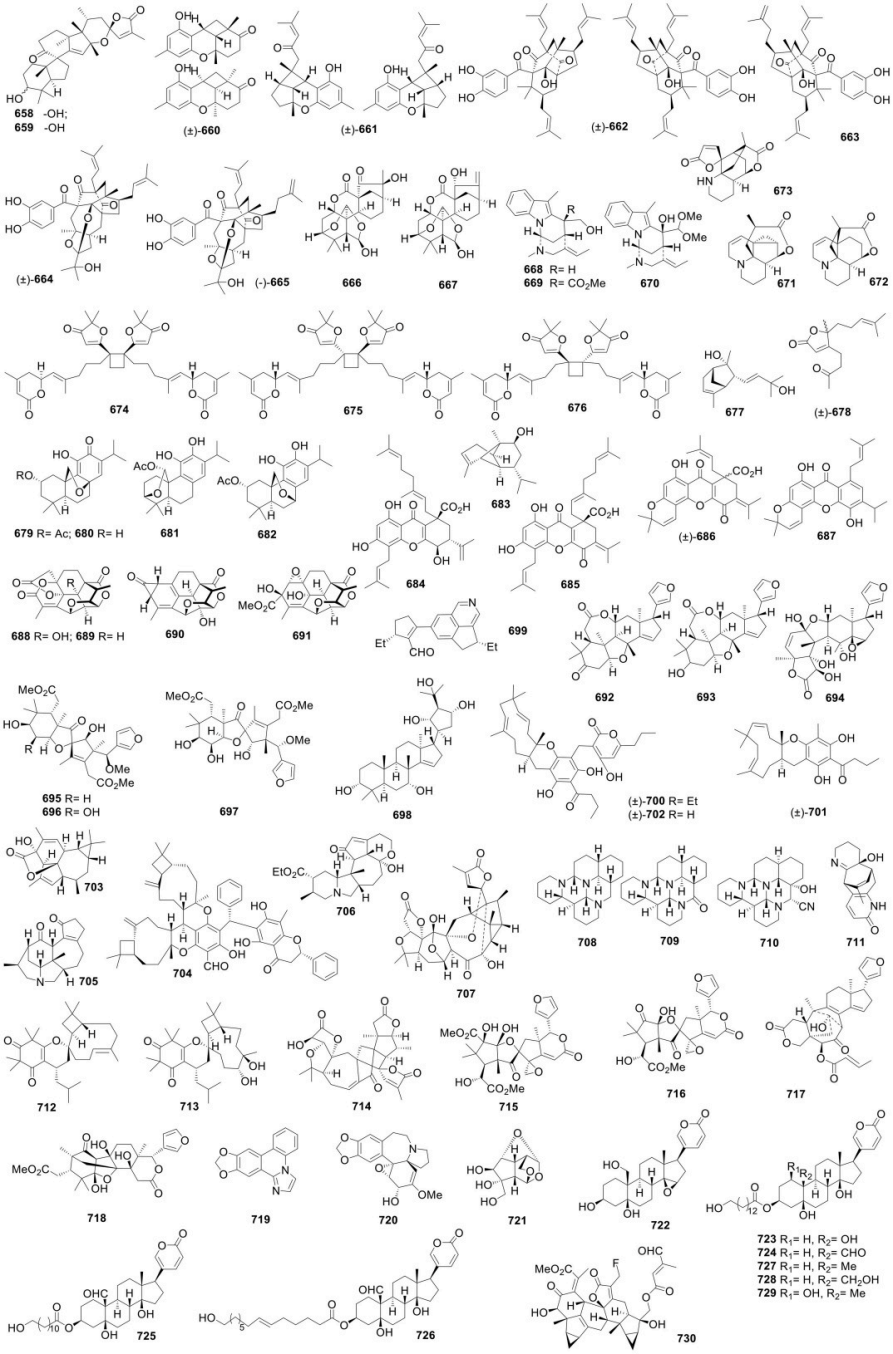
Structures of compounds **658-730**.

Four new iridals with an α-terpineol moiety resulting from cyclization of the homofarnesylside chain, polycycloiridals A–D (**650**-**653**), were from the rhizomes of *Iris tectorum* (Zhang et al., 2015a). Diterpenoid randainins A–D (**654**-**657**) with a *trans*-fused 7/5 or 5/7 ring system were isolated from leaves and twigs of *Callicarpa randaiensis* (Nantou, Taiwan, China) (Cheng et al., 2015). Research into *Abies chensiensis* led to the discovery of triterpenoid spirochensilides A (**658**) (Figure 11) and B (**659**) possessing a 8,10-cyclo-9,10-seco skeleton (Zhao et al., 2015b). Two pairs of enantiomeric meroterpenoids, (±)-rhodonoids A ((±)-**660**) and B ((±)-**661**) with a unique 6/6/6/4 ring system, were isolated from *Rhododendron capitatum* (Liao et al., 2015a). *Garcinia multiflora* was the source of (±)-garcimulins A ((±)-**662**) and B (**663**) as well as (±)-garmultin A ((±)-**664**) and (–)-garmultin B ((–)-**665**). (±)-Garcimulins A ((±)-**662**) and B (**663**) included a tetracyclo[5.4.1^1, 5^.1.0^9, 13^]tridecane skeleton (Fan et al., 2015a), while (±)-garmultin A ((±)-**664**) and (–)-garmultin B ((–)-**665**) coupled a 2,11-dioxatricyclo[4.4.1.0^3, 9^] undecane and a tricyclo [4.3.1.0^3, 7^]decane (Tian et al., 2016a). Thirteen enmein-type *ent*-kaurane diterpenoids were obtained from aerial parts of *Isodon phyllostachys* (Sichuane, China), among which phyllostacins J (**666**) and K (**667**) were the first examples of 3,20:6,20-diepoxyenmein-type *ent*-kauranoids (Yang et al., 2016e). Alstoscholarisines H–J (**668**–**670**), three new monoterpenoid indole alkaloids with a new skeleton created by forming a C-3/N-1 bond, were obtained from the leaves *Alstonia scholaris* that was registered as an investigational new botanical drug (No. 2011L01436) and approved for clinical trials (phases I and II) by the China Food and Drug Administration (CFDA) (Pan et al., 2016b).

Annotinolides A–C (**671**-**673**), three novel 7,8-seco-lycopodanoid 8,5-lactones, were afforded by *Lycopodium annotinum* (Tang et al., 2016b). *Aphanamixis grandifolia* (Hainan, China) produced three novel diterpenoids dimers, aphadilactones E–G (**674**-**676**) (Zhang et al., 2016b). Artaboterpenoid A (**677**) and (±)-artaboterpenoid B ((±)-**678**), two new bisabolene-derived sesquiterpenoids, were obtained from the roots of *Artabotrys hexapetalus* (Xi et al., 2016). Six new compounds were identified from *Salvia plebeian*, among which compounds **679**–**682** were diterpenoids bearing an oxygen bridge while compound **683** was a copane-type sesquiterpenoid with a bridged tricyclic framework (Xu et al., 2016b). Oliganthic acids A–D (**684**-**687**), four prenylated isopropyl xanthone derivatives, were isolated from the leaves of *Garcinia oligantha* (Tang et al., 2016c). Cephalotanins A–D (**688**–**691**), four polycyclic norditerpenoids with highly rigid ring systems, were obtained from *Cephalotaxus sinensis* (Guangxi, China) (Xu et al., 2016a).

Three new ring B-seco limonoids, ciliatonoids A–C (**692**-**694**), were produced by *Toona ciliate*. Ciliatonoids A (**692**) and B (**693**) featured an unprecedented limonoid architecture (Liu et al., 2016a). *Cipadessa cinerascens* was the source of four limonoids with spirocyclic skeletons, cipacinoids A–D. Cipacinoids A–C (**695**-**697**) were the first examples of 17*S*- limonoids (Yu et al., 2016b). Phytochemical investigation of the stems of *Picrasma quassioides* (Jiangxi, China) led to the isolation of picraquassin A (**698**) featuring an unprecedented 21,24-cycloapotirucallane skeleton (Xu et al., 2016c). Delavatine A (**699**), a cyclopenta[*de*]isoquinoline alkaloid, was afforded by *Incarvillea delavayi* (Yunnan, China) (Zhang et al., 2016n). *Dryopteris championii* (Hainan, China) produced (±)-drychampones A–C ((±)-**700**, (±)-**701**, and (±)-**702**), three novel meroterpenoids constructed by a 11/6/6 ring system and a pyronone moiety (Chen et al., 2016d). Euphorikanin A (**703**), characterized by an unusual 5/6/7/3-fused tetracyclic ring skeleton, was obtained from the roots of *Euphorbia kansui* (Fei et al., 2016). Guajavadimer A (**704**), a dimeric meroterpenoid featuring two caryophyllenes, one benzylphlorogulcinol, as well as one flavonone-fused complicated stereochemical skeleton, was isolated from the leaves of *Psidium guajava* (Li et al., 2016a). *Daphniphyllum himalense* produced himalensines A (**705**) and B (**706**) with unprecedented carbon skeletons (Zhang et al., 2016c).

*Schisandra lancifolia* was the source of lancolide E (**707**) that possessed a complex tetracyclo [5.4.0.0^2, 4^.0^3, 7^]undecane-bridged system (Shi et al., 2016). Three new alkaloids with the heterohexacyclic skeleton, myritonines A–C (**708**-**710**), were afforded by *Myrioneuron tonkinensis* (Guangxi, China) (Li et al., 2016i). A new C_16_N_2_ lycopodium alkaloid, affordedphlefargesiine A (**711**), was isolated from *Phlegmariurus fargesii* (Meng et al., 2016b). Rhodomentones A (**712**) and B (**713**), two new meroterpenoids bearing a caryophyllene-conjugated oxa-spiro teradecadiene skeleton, were afforded by *Rhodomyrtus tomentosa* (Jiangxi, China) (Liu et al., 2016b). A schinortriterpenoid possessing a tricyclo[5.2.1.0^1, 6^]decane-bridged system, schincalide A (**714**), was obtained from the stems and leaves of *Schisandra incarnate* (Hubei, China) (Zhou et al., 2016d). Fruits of *Trichilia connaroides* contained spirotrichilins A (**715**) and B (**716**), two novel limonoids with a spiro (cyclopenta[b]furan-2,1′-cyclopentane) system (An et al., 2016a). Another two novel limonoids, trichiconlides A (**717**) and B (**718**), were also isolated from fruits of *T*. *connaroides* (An et al., 2016c). The first naturally occurring imidazo[1,2-f]phenanthridine alkaloid, zephycandidine A (**719**), was produced by *Zephyranthes candida* (Hubei,China) (Zhan et al., 2016). A new cephalotaxus alkaloid, cephalolancine A (**720**), was isolated from *Cephalotaxus lanceolata* (Ni et al., 2016). Valeriridoid P (**721**), an unusual iridoid containing a two oxo-bridge fragment, was discovered from the roots of *Valeriana officinalis* var. *latiofolia* (Guizhou, China) (Wang et al., 2015c).

## Terrestrial animals

A total of 90 new NPs was isolated from terrestrial insects and other animals. Of these, 37.8% compounds exhibited various bioactivities, much higher than the average of 28.4%. Nine NPs reported in two references with significant bioactivities are listed in this section.

The eggs of toad *Bufo bufo gargarizans* afforded two novel 19-norbufadienolides, a new 14,15-epoxy bufadienolide as well as eight rare bufadienolide-fatty acid conjugates, among which compounds **722**-**729** displayed cytotoxic activities against MCF-7 and MDA-MB-231 cells with IC_50_ ranges of 0.027–0.315 and 0.05–1.96 μM, respectively (Zhang et al., 2016g). Twelve new sesquiterpenoid dimmers, fortunilides A–L, were isolated from the malaria parasite *Plasmodium falciparum* (Guangxi, China). Fortunilide A (730) exhibited *P. falciparum* growth inhibition with an IC_50_ of 5.2 nM (artemisinin, 4.0 nM) (Zhou et al., 2017).

## Conclusion

This review covers the NPs literatures from 2015 to 2016 by chemists from China and describes 6,944 new NPs reported from 1,985 papers. The average number of new compounds per paper is 3.5. Compared with 2015, the number of new NPs decreased by 22% in 2016 from 3,891 to 3,053. Elucidation of new compounds and assessment of their bioactivities along with methods for their syntheses, corrections of stereochemistry, and mechanistic as well as biosynthetic studies were two major areas of NPs research. However, the discovery of new compounds was undoubtedly the basis of all studies. In recent years, based on the large numbers of identified compounds, chemists increasingly began to focus on the other aspects described above, resulting in the decline of the identification of new compounds in 2016.

According to the survey of the literature from 2015 to 2016 (Supplementary Table 1), 730 new NPs out of 6,944 identified compounds possessed significant bioactivity and/or a novel skeleton. Their detailed information is compiled in this review.

**Figure 12:**
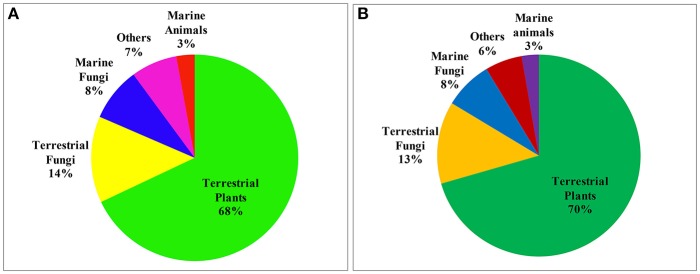
Biological sources of publications **(A)** and new NPs **(B)** in China (2015–2016).

According to our review, terrestrial plants (number of publications/compounds, 1,348/4,895), terrestrial fungi (269/910), and marine fungi (167/535) were the main sources of new NPs at 68%,14%, and 8% in terms of total papers referenced (Figure 12A) and 70%, 13%, 8% in terms of total compounds (Figure 12B), respectively.

**Figure 13:**
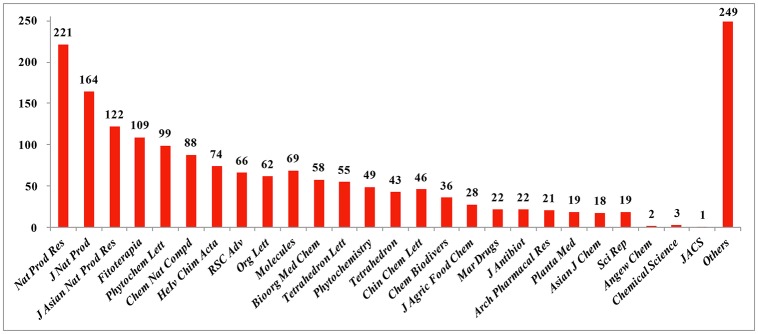
Numbers of NPs articles from China published in each journal (2015–2016).

**Figure 14:**
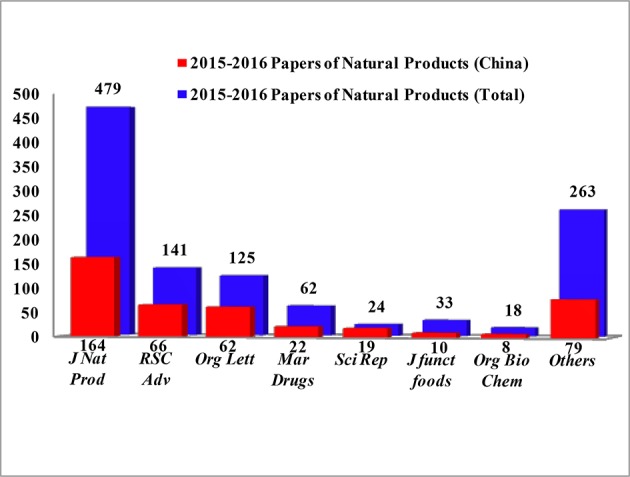
Numbers of new NPs papers with an impact factor of greater than 3.0 (2015–2016).

A total of 1,765 papers were published in over 110 international journals in the past two years. Figure 13 shows the total number of NP articles from China sorted by journal. *Natural Product Research* (publications/percentage in total publications of China, 221/12%) was the first choice for chemists from China to publish their newly discovered NPs followed by *Journal of Natural Products* (164/9%), *Journal of Asian Natural Products Research* (122/7%), and *Fitoterapia* (109/6%). Notably, more than a quarter of articles (457 articles) were published in 38 journals with impact factors ≥3.0. The *Journal of Natural Products*, as a recognized journal in the field of NPs, undoubtedly attracted the most contributions (164 articles) and was followed by *RSC Advances* (66 articles), *Organic Letters* (62 articles), and *Marine Drugs* (22 articles); among all articles related to new NPs published in these journals, 34, 47, 50, and 35% were contributed by Chinese NPs chemists, respectively (Figure 14).

**Figure 15:**
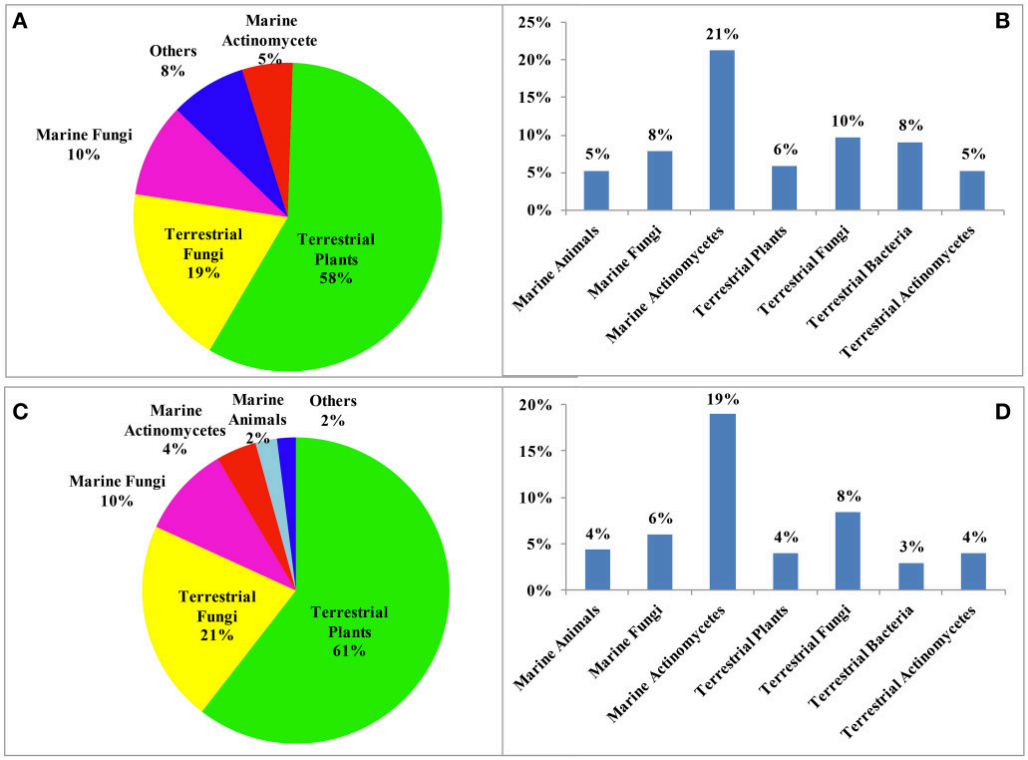
Occurrence of papers describing novel skeletal NPs **(A)**; papers related to novel skeletons out of the same biological source **(B)**; biological sources of novel skeletal NPs **(C)**; occurrence of novel skeletal compounds out of the same biological source **(D)**.

These results indicate that chemists from China play an irreplaceable role in NPs research and have made great contributions to the discovery of new compounds. It is also worth noting that there were only a few articles relevant to new NPs published in top chemistry journals with significant worldwide influence, such as *Angewandte Chemie International Edition* (China/total, 2/27), *Chemical Science* (3/4), and *Journal of the American Chemical Society* (2/5) (Figure 13). This review of the current progress of NPs chemistry shows that a multidisciplinary approach is henceforth an inevitable trend. Strengthening interdisciplinary cooperation is essential and biosynthesis, bioinformatics, and pharmacology as well as computer-aided technologies should be integrated to advance the discovery of more lead compounds with the potential to be developed into drugs. Currently, chemists from China face the challenging task of value mining of identified NPs rather than simply elucidating the structures of novel compounds. Molecules with unprecedented skeletons always attract attention and interest in the NPs research field. In 2015–2016, 134 papers (7% in total) were published and reported 352 NPs (5% in total) with novel skeletons. The dominant biological sources of new skeletal NPs were terrestrial plants (papers/percentage in 134 papers, 79/58%), terrestrial fungi (26/19%), and marine fungi (13/10%) (Figure 15A), from which 213 (percentage in all novel skeletons, 61%), 75 (21%), and 34 (10%) new skeletal NPs were isolated, respectively (Figure 15C). However, the percent occurrence of novel skeletons out of each biological source was slightly different. Marine actinomyctes possessed the highest occurrence of novel skeletons in 21% of papers (Figure 15B) and 19% of NPs (Figure 15D) followed by terrestrial fungi (10%, 8%), terrestrial bacteria (8%, 3%), and marine fungi (8%, 6%). This indicated that the microorganism, especially marine actinomyctes, was the major biological source of new skeletal compounds in China with great potential for research, and deserved to be extensively studied.

**Figure 16:**
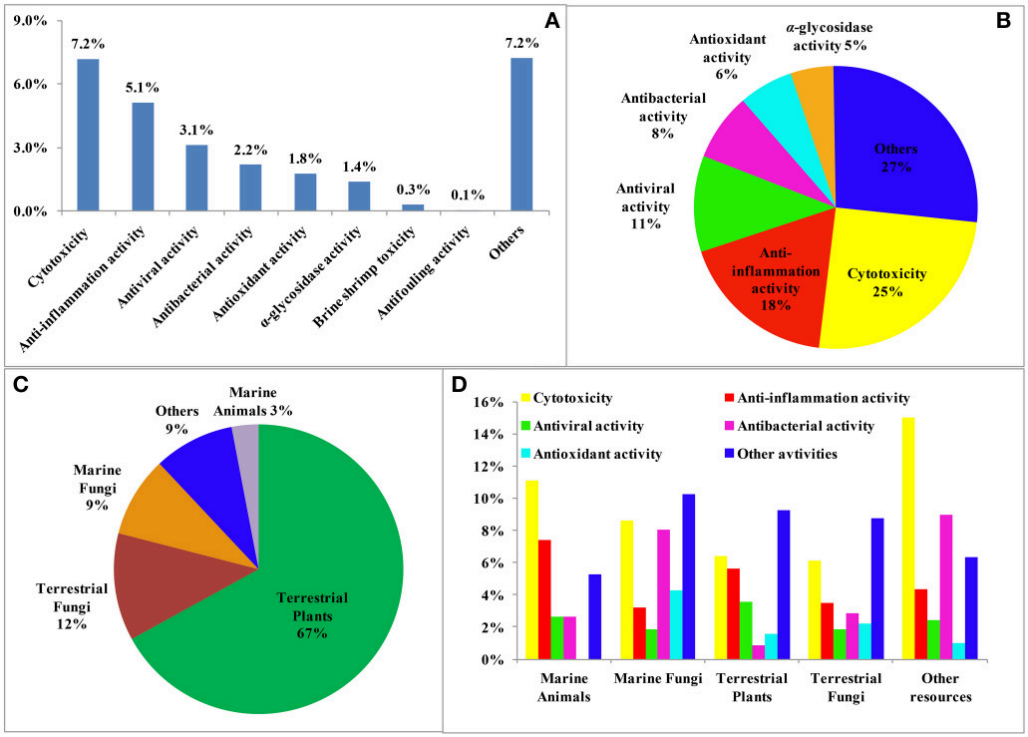
The percent of each type bioactive NPs out of all NPs **(A)** and all bioactive NPs **(B)**; biological sources of bioactive NPs **(C)** and distribution between biological sources and bioactivities **(D)**.

The results showed that 28% of new NPs displayed a variety of bioactivities. The dominant bioactivities were cytotoxicity, anti-inflammatory activity, and antiviral activity with 7%, 5%, and 3% ratios in all NPs (Figure 16A) and 25%, 18%, and 11% ratios in all bioactive NPs (Figure 16B), respectively. Biological sources were also taken into consideration when compiling the data. A total of 68% of the bioactive compounds were isolated from terrestrial plants followed by terrestrial and marine fungi with 12% and 9% ratios, respectively (Figure 16C). The distribution between biological sources and bioactivities was also analyzed (Figure 16D). Cytotoxicity and anti-inflammatory activity were the major bioactivities of NPs isolated from marine animals and terrestrial plants, whereas cytotoxic and antibacterial activities were dominant for those from marine fungi. This analysis may serve as a guideline for purposeful investigation of bioactive NPs.

As reviewed in this article, China had made enormous strides in NPs chemistry development and contributed substantial numbers of promising bioactive molecules to drug research in the past two years. We believe that there will continue to be a steady development of NPs research in China, and large numbers of more extensive studies as well as innovative approaches will be available in the near future.

## Author contributions

All authors listed have made a substantial, direct and intellectual contribution to the work, and approved it for publication.

### Conflict of interest statement

The authors declare that the research was conducted in the absence of any commercial or financial relationships that could be construed as a potential conflict of interest.
