# Supplementary material for: Natural Products Research in China From 2015 to 2016
Source: Front Chem. 2018 Mar 20;6:45. doi: 10.3389/fchem.2018.00045 (PMC5869933; doi:10.3389/fchem.2018.00045)
Supplement: Supplementary file 1 [file Table1.docx]

Supplementary Material

Natural Products Research in China from 2015 to 2016

**Haishan Liu, Guoliang Zhu, Yaqin Fan, Yuqi Du, Mengmeng Lan, Yibo Xu, Weiming Zhu***

*** Correspondence:** Weiming Zhu: [weimingzhu@ouc.edu.cn](mailto:weimingzhu@ouc.edu.cn)

**Supplementary Table 1**. NPs with Significant Bioactivity and/or a Novel Skeleton Isolated by Chinese Chemists (2015–2016)

| **Compound** | **Producer** | **Environment source** | **Bioavtivity^a^** | **Ref.** |
| --- | --- | --- | --- | --- |
| **1**, **2** | *Microbacterium sediminis* YLB-01(T) | Unknown deep sea | Cytotoxicity | (Liu et al., 2015b) |
| **3** | *Micromonospora* sp. FIM02-523 | Sediment, Fujian province, China | Cytotoxicity | (Lin et al., 2016b) |
| **4** | *Streptomyces* sp. LS298 | *Gelliodes carnosa*, Lingshui Bay, Hainan province, China | Cytotoxicity | (Zhen et al., 2015) |
| **5**, **6** | *Streptomyces pactum* SCSIO 02999 | Unknown sea area | Cytotoxicity | (Saha et al., 2017) |
|  |  |  |  |  |
| **7**, **8** | *Streptomyces sp*. OUCMDZ-3434 | *Enteromorpha prolifera*, Zhanqiao Beach, China | *α*-glucosidase inhibitory activity | (Chen et al., 2016g) |
| **9**–**11** | *Streptomyces* sp. LZ35 | Unknown source | / | (Li et al., 2015f) |
|  |  |  |  |  |
| **12** | *Streptomyces griseus* strain M268 | Sediment, Kiaochow Bay, China | / | (Xie et al., 2016b) |
| **13**–**16** | *Streptomyces* sp. SCSGAA 0027 | *Melitodes squamata*, South China Sea | / | (Nong et al., 2016) |
| **17**, **18** | *Streptomyces* sp. HDN-10-293 | Unknown sponge | / | (Che et al., 2016) |
| **19** | *Streptomyces* sp. 219807 | Mangrove soil, Hainan province, China | Cytotoxicity | (Han et al., 2016b) |
| **20** | *Streptomyces sp. CHQ-64* | Mangrove conservation area, Guangdong province, China | / | (Che et al., 2015) |
|  |  |  |  |  |
| **21** | *Curvularia* sp. IFB-Z10 | White croaker | Anti-inflammation | (Han et al., 2016c) |
| **22**–**26** | *Acremonium* sp. | Deep-sea sediments, South Atlantic Ocean | Anti-inflammation | (Cheng et al., 2016) |
| **27**–**31** | *Aspergillus ochraceopetaliformis* SCSIO 05702 | Sediment, Chinese Antarctic station | Antiviral activity | (Wang et al., 2016b) |
|  |  |  |  |  |
| **32**–**37** | *Engyodontium album* strain LF069 | *Cacospinga scalaris*, Limski Fjord, Croatia | Antibacterial activity | (Wu et al., 2016) |
| **38** | *Pleosporales* sp. CF09-01 | Sediment, Bohai Sea, China | Antibacterial activity | (Cao et al., 2016a) |
| **39** | *Penicillium aculeatum* SD-321 | deep-sea sediment, South China sea | Antibacterial activity | (Li et al., 2015h) |
| **40**–**44** | *Penicillium sp.* F23-2 | Unknown deep sea | Antibacterial activity | (Guo et al., 2015a) |
|  |  |  |  |  |
|  |  |  |  |  |
|  |  |  |  |  |
| **45** | *Penicillium adametzioides* AS-53 | Sponge, Hainan Island, China | Brine shrimp lethality Antibacterial activity | (Liu et al., 2015i) |
| **46**, **47** | *Spiromastix* sp. MCCC 3A00308 | Sediment at depth of 2869 m, South Atlantic Ocean | Antibacterial activity | (Niu et al., 2015) |
|  |  |  |  |  |
| **48**, **49** | *Aspergillus* sp. SCSIO XWS02F40 | *Callyspongia* sp, Guangdong province, China | Antiviral activity | (Tian et al., 2015c) |
|  |  |  |  |  |
|  |  |  |  |  |
|  |  |  |  |  |
|  |  |  |  |  |
|  |  |  |  |  |
| **50** | *Arthrinium arundinis* ZSDS1-F3 | Sponge, Xisha Islands, China | AchE inhibitory activity | (Wang et al., 2015e) |
| **51**, **52** | *Pestalotiopsis* sp. ZJ-2009-7-6 | *Sarcophyton* sp., Yongxing Island, South China Sea | Antifouling activity | (Xing et al., 2016) |
| **53**–**57** | *Talaromyces islandicus* EN-501 | Marine red alga *Laurencia okamurai*, Shandong province, China | Antioxidant activity | (Li et al., 2017) |
| **58** | *Penicillium citrinum* | Sediment, Langqi Island, Fujian province, China | / | (Liu et al., 2015d) |
| **59**–**63** | *Gliomastix* sp. ZSDS1-F7-2 | *Phakellia fusca* Thiele, South China Sea | / | (Zhang et al., 2015h) |
| **64**–**66** | *Pseudallescheria ellipsoidea* F42-3 | *Lobophytum crassum*, Sanya National Coral Reef Reserve, Hainan province, China | / | (Liu et al., 2015e) |
|  |  |  |  |  |
| **67** | *Penicillium chrysogenum* SCSIO41001 | Deep-sea sediment, Indian Ocean | / | (Chen et al., 2016f) |
| **68**, **69** | *Cladosporium cladosporioides* HDN14-342 | Sediment, Indian Ocean | / | (Zhang et al., 2016m) |
| **70**–**72** | Mutated strain *Emericella variecolor* XSA-07-2 | Unknown sponge | / | (Long et al., 2016) |
| **73**, **74** | *Aspergillus flavipes* | Unknown source | / | (Zhu et al., 2016) |
|  |  |  |  |  |
| **75**, **76** | *Paraconiothyrium sporulosum* YK-03 | Sea mud, Bohai Bay, China | / | (Zhang et al., 2016d) |
|  |  |  |  |  |
|  |  |  |  |  |
| **77** | *Aspergillus versicolor* HDN 1009 | Guangdong province, China | Cytotoxicity | (Wu et al., 2015a) |
| **78**, **79** | *Penicillium janthinellum* HDN13-309 | Hainan province, China | Cytotoxicity | (Zhu et al., 2017) |
| **80, 81** | *Neosartorya udagawae HDN13-313* | Aricennia marina, Hainan province, China | Antiviral activity | (Yu et al., 2016a) |
| **82** | *Penicillium brocae MA-231* | Avicennia marina, Hainan Island, China | Antibacterial activity | (Meng et al., 2015b) |
| **83–85** | *Penicillium brocae MA-231* | Avicennia marina, Hainan Island, China | Antibacterial activity | (Meng et al., 2015c) |
| **86, 87** | *Penicillium brocae MA-231* | Avicennia marina, Hainan Island, China | / | (Meng et al., 2016a) |
| **88** | *Stemphylium sp. 33231* | Brguiera sexangula var. rhynchopetala, South China Sea | Antibacterial activity | (Zhou et al., 2015d) |
| **89, 90** | *Penicillium sp. HN29-3B1* | Cerbera manghas, Dongzhaigang Mangrove National Nature Reserve, Hainan Island, China | α-glucosidase inhibitory activity | (Liu et al., 2015j) |
| **91** | *Aspergillus sp. 16-5B* | Sonneratia apetala, Dongzhaigang Mangrove National Nature Reserve, Hainan Island, China | α-glucosidase inhibitory activity | (Liu et al., 2015h) |
| **92–94** | *Aspergillus sp. 085242* | Acanthus ilicifolius, Shankou Mangrove National Nature Reserve, Guangxi Zhuang Autonomous Region, China | α-glucosidase inhibitory activity | (Xiao et al., 2016b) |
| **95** | *Talaromyces amestolkiae YX1* | Kandelia obovata, Zhanjiang Mangrove Nature Reserve, Guangdong province, China | α-glucosidase inhibitory activity | (Chen et al., 2016e) |
| **96** | *Eurotium rubrum MA-150* | Mangrove-derived rhizospheric soil, Andaman Sea coastline, Thailand | Brine shrimp lethality | (Meng et al., 2015a) |
| **97, 98** | *Aspergillus terreus H010* | Kandelia obovata | / | (Liu et al., 2016e) |
| **99–101** | *Mucor irregularis QEN-189* | Rhizophora stylosa, Hainan Island, China | / | (Gao et al., 2016b) |
| **102**–**104** | *Dysidea* sp. | South China Sea, at a depth of 10 m | Cytotoxicity NF-κB inhibitory activity | (Jiao et al., 2016) |
| **105**–**107** | *Dysidea fragilis* | South China Sea | Anti-inflammation | (Jiao et al., 2015a) |
| **108** | *Carteriospongia foliascens* | Dongluoxigu Island, China | Cytotoxicity | (Cao et al., 2015a) |
| **109** | *Sinularia verruca* van Ofwegen | Ximao Island, Hainan province, China | Antiviral activity | (Yuan et al., 2016) |
| **110**, **111** | *Subergorgia rubra* | South China sea | Antibacterial activity | (Sun et al., 2015c) |
| **112** | *Fascaplysinopsis reticulata* | South China Sea | / | (Wang et al., 2015i) |
| **113** | *Lysobacter enzymogenes* | University of Nebraska Lincoln, U.S. | Cytotoxicity | (Xu et al., 2015b) |
| **114** | *Bradyrhizobium* sp. BTAi1 (ATCC BAA-1182) |  | / | (Chen et al., 2016c) |
| **115**, **116** | *Bacillus subtilis* fmb60 | Jiangsu province, China | Antibacterial activity | (Yang et al., 2016) |
| **117** | *Streptomyces* sp. CC8-201 | Karst cave soil sample, Chongqing, China | Cytotoxicity | (Jiang et al., 2015b) |
| **118** | *Nocardiopsis lucentensis* DSM 44048 | Saltmarsh soil sample | Cytotoxicity | (Sun et al., 2015a) |
| **119** | *Streptomyces chattanoogensis* L10 (CGMCC 2644) | Unknown source | Cytotoxicity | (Zhou et al., 2015e) |
| **120** | Mutant strain *Streptomyces avermectinius* | Unknown source | Cytotoxicity | (Sun et al., 2015b) |
| **121** | Mutant strain *Streptomyces* sp. LZ35 | Unknown source | Cytotoxicity | (Li et al., 2015g) |
| **122**, **123** | *Streptomyces phytohabitans* HBERC-20821 | Soil, Wawushan Hill, Sichuan province, China | Antibacterial activity | (Wan et al., 2015) |
| **124** | *Streptomycetes* no. 8 | Qinling Mountains, Shaanxi province, China | *α*-glucosidase inhibitory activity | (Wei et al., 2016a) |
| **125**–**127** | Engineered strain *Streptomyces avermitilis* MHJ1011 | Unknown source | Acaricidal activity | (Pan et al., 2016a) |
| **128**, **129** | *Streptomyces* sp. FXJ1.532 | Red soil, Jiangxi province, China | / | (Guo et al., 2015b) |
| **130** | Engineered strain *Streptomyces coelicolor* YF11 | Unknown source | / | (Yang et al., 2015a) |
| **131** | *Streptomyces* sp. KIB-H033 | *Camellia sinensis* | / | (Yan et al., 2016) |
| **132** | *Aspergillus clavatus* | *Tripterygium hypoglaucum* | Cytotoxicity | (Wang et al., 2015b) |
| **133**–**137** | *Pleurotus cystidiosus* | Qingyun mountains, Fujian province, China | Cytotoxicity | (Zheng et al., 2015b) |
| **138** | *Myrothecium roridum* A553 | *Pogostemon cablin*, Guangdong province, China | Cytotoxicity | (Liu et al., 2016c) |
| **139** | Unknown fungus FR02 | *Ficus carica*, Qinling Mountain, Shaanxi province, China | Cytotoxicity Antibacterial activity | (Ma et al., 2016d) |
| **140**–**143** | *Acremonium persicinum* SC0105 | Soil, Dinghu Mountain Biosphere Reserve, Guangdong province, China | / | (Wu et al., 2015d) |
| **144**–**146** | *Acremonium persicinum* SC0105 | Soil, Dinghu Mountain Biosphere Reserve, Guangdong province, China | Anti-inflammation | (Wu et al., 2015e) |
| **147**, **148** | *Aspergillus terreus* PR-P-2 | *Camellia sinensis* var. assamica, Yunnan province, China | Anti-inflammation | (Guo et al., 2016) |
| **149**–**151** | *Penicillium purpurogenum* MHz 111 | Soil, Heilongjiang province, China | Anti-inflammation | (Sun et al., 2016b) |
| **152**–**155** | *Periconia* sp. F-31 | *Annona muricata*, Hainan province, China | Anti-inflammation | (Liu et al., 2016d) |
| **156** | *Periconia* sp. F-31 | *Annona muricata*, Hainan province, China | / | (Zhang et al., 2015b) |
| **157**–**160** | *Ganoderma sinense* | Unknown source | Anti-inflammation | (Wang et al., 2016d) |
| **161**–**164** | *Fomes officinalis* | Xinjiang Uyghur Medicine Hospital, Xinjiang province, China | Anti-inflammation | (Han et al., 2016a) |
| **165** | *Aspergillus ochraceus* KM007 | an unidentified tree near Lake Fuxian, Yunnan province, China | Antibacterial activity | (Chang et al., 2016) |
| **166** | *Grifola frondosa* | Wuyi Mountain, Fujian province, China | Antibacterial activity | (He et al., 2016) |
| **167** | *Fusarium sp.* | *Ficus carica,* Qinling Mountain, Shaanxi province, China | Antibacterial activity | (Liang et al., 2016) |
| **168**–**170** | *Aspergillus flavipes* PJ03-11 | Wetland mud, Panjin Red Beach National Nature Reserve, Liaoning province, China | *α*-glucosidase inhibitory activity | (Zhang et al., 2016f) |
| **171**, **172** | *Aspergillus flavipes* PJ03-11 | Wetland mud, Panjin Red Beach National Nature Reserve, Liaoning province, China | *α*-glucosidase inhibitory activity | (Zhang et al., 2016e) |
| **173**–**177** | *Ganoderma lucidum* | Kingsci Biotechnology Co. Ltd., China | Cytotoxicity *α*-glucosidase inhibitory activity | (Zhao et al., 2015c) |
| **178**, **179** | *Ganoderma leucocontextum* | Nyingchi, Tibet, China | *α*-glucosidase inhibitory activity | (Wang et al., 2016c) |
| **180**–**188** | *Ganoderma leucocontextum* | Tibet plateau, China | *α*-glucosidase inhibitory activity | (Wang et al., 2015g) |
| **189**–**198** | *Hericium erinaceus* | Tibet plateau, China | *α*-glucosidase inhibitory activity | (Wang et al., 2015f) |
| **199** | *Aspergillus terreus* 3.05358 | China General Microbiological Culture Collection Center | *α*-glucosidase inhibitory activity | (Shan et al., 2015) |
| **200** | *Aspergillus terreus* | Soil of Yangzi River, Hubei province, China | BACE1 inhibitory activity | (Qi et al., 2016) |
| **201**, **202** | *Ganoderma boninense* | Hainan province, China | Anti-*Plasmodium falciparum* | (Ma et al., 2015c) |
| **203**–**205** | *Sordaria macrospora* | *Ilex cornuta* | Antioxidant activity | (Li et al., 2016h) |
| **206**–**212** | *Boreostereum vibrans* | Kunming Botanical Garden, China | Pancreatic lipase inhibitoryory activity | (Chen et al., 2016b) |
| **213**–**215** | *Cunninghamella elegans* AS 3.2028 | China General Microbiological Culture Collection Center, Beijing, China | Platelet aggregation inhibitory activity | (Tian et al., 2016b) |
| **216**, **217** | *Phoma* sp. | Soil, Qinghai-Tibetan plateau, China | / | (Zhang et al., 2015i) |
| **218** | *Ganoderma sinensis* | Unknown source | / | (Luo et al., 2015b) |
| **219** | *Ganoderma applanatum* | Unknown source | / | (Luo et al., 2015a) |
| **220**, **221** | *Ganoderma applanatum* | Unknown source | / | (Luo et al., 2016a) |
| **222** | *Ganoderma applanatum* | Unknown source | / | (Li et al., 2016f) |
| **223**, **224** | *Chaetomium globosum* TW1-1 | *Armadillidium vulgare* | / | (Chen et al., 2015a) |
| **225**–**231** | *Aspergillus tubingensis* KJ-9 | Unknown source | / | (Tang et al., 2015) |
| **232**–**234** | *Gloeophyllum abietinum* | Unknown source | / | (Han et al., 2015) |
| **235**–**238** | *Neonectria* sp. | Soil, Qinghai-Tibetan plateau, China | / | (Ren et al., 2015b) |
| **239**–**244** | Mutant strain *Daldinia eschscholzii* | *Tenodora aridifolia* | / | (Tian et al., 2015b) |
| **245**–**247** | *Periconia* sp. | *Parmelia* sp., Changbai Mountain, Jilin province, China | / | (Wu et al., 2015c) |
| **248**, **249** | *Pestalotiopsis fici* | *Camellia sinensis* (Theaceae), Zhejiang province, China | / | (Liu et al., 2015c) |
| **250**, **251** | *Stereum hirsutum* | Tibet, China | / | (Qi et al., 2015) |
| **252** | *Chaetomium seminudum* | Shaanxi Institute of Microbiology, China | / | (Li et al., 2015b) |
| **253**, **254** | *Pestalotiopsis theae* N635 | *Camellia sinensis*, Zhejiang province, China | / | (Liu et al., 2016f) |
| **255**–**260** | *Hypocrea* sp. EC1-35 | *Septobasidium*-infected *Serrataspis* sp. | / | (Ren et al., 2016) |
| **261**–**266** | *Emericella nidulans* HDN12-249 | *Tamarix chinensis* Lour, Laizhou Bay, China | / | (Zhou et al., 2016a) |
| **267**–**269** | *Lecanicillium* sp. PR-M-3 | Unknown source | / | (Wang et al., 2016h) |
| **270** | *Daldinia eschscholzii* IFB-TL01 | *Tenodera aridifolia* | / | (Zhang et al., 2016a) |
| **271**–**276** | *Hypericum sampsonii* | Dabie Mountain, Hubei province, China | Cytotoxicity | (Zhu et al., 2015a) |
| **277**, **278** | *Hypericum subsessile* | Unknown source | Cytotoxicity | (Liao et al., 2015b) |
| **279** | *Maytenus austroyunnanensis* | Yunnan province, China | Cytotoxicity | (Tan et al., 2016) |
| **280** | *Carpesium abrotanoides* L. | Unknown source | Cytotoxicity Antibacterial activity | (Wang et al., 2015d) |
| **281** | *Tupistra chinensis* | Shennongjia Forest, Hubei province, China | Cytotoxicity | (Xiao et al., 2015) |
| **282** | *Trichilia americana* | XTBG, Mengla country, Yunnan province, China | Cytotoxicity | (Ji et al., 2015) |
| **283** | *Illicium merrillianum* | Gongshan county, Yunnan province, China | Cytotoxicity | (Tian et al., 2015a) |
| **284**, **285** | *Tripterygium wilfordii* | Taining, Fujian province, China | Cytotoxicity | (Wang et al., 2015a) |
| **286**, **287** | *Streptocaulon juventas* | Yunnan province, China | Cytotoxicity | (Ye et al., 2015) |
| **288**, **289** | *Aglaia odorata* | Xishuangbanna, Yunnan province, China | Cytotoxicity | (An et al., 2015) |
| **290** | *Aglaia odorata* | Longzhou County, Guangxi Zhuang Autonomous Region | Cytotoxicity | (Peng et al., 2016a) |
| **291**, **292** | *Aglaia odorata* | Unknown source | Cytotoxicity | (Liu and Xu, 2016) |
| **293** | *Isodon eriocalyx* var. *laxiﬂora* | Yunnan province, China | Cytotoxicity | (Wang et al., 2015g) |
| **294** | *Croton tiglium* | Sichuan province, China | Cytotoxicity | (Zhang et al., 2016j) |
| **295**–**297** | *Annona squamosa* | Guangdong province, China | Cytotoxicity | (Miao et al., 2016) |
| **298** | *Taxus wallichiana.* var. *mairer* | Jiangsu Yew pharmaceutical Co., Ltd., China | Cytotoxicity | (Wang et al., 2016f) |
| **299**, **300** | *Physalis pubescens* L. | Liaoning province, China | Cytotoxicity | (Xia et al., 2016) |
| **301** | *Aglaia perviridis* | Xishuangbanna, Yunnan province, China | Cytotoxicity | (An et al., 2016b) |
| **302** | *Carpesium cernuum* | Guizhou province, China | Cytotoxicity | (Liu et al., 2016g) |
| **303**–**308** | *Chloranthus sessilifolius* | Fengqi Mountains, Sichuan province, China | Anti-inflammation | (Wang et al., 2015h) |
| **309**, **310** | *Murraya exotica*. | Unknown source | Anti-inflammation | (Liu et al., 2015a) |
| **311**–**315** | *Inula britannica* | Unknown source | Anti-inflammation | (Zhang et al., 2015l) |
| **316**–**324** | *Ginkgo biloba* leaves | Chongming Island, China | Anti-inflammation | (Ma et al., 2016a) |
| **325**, **326** | *Corydalis hendersonii* | Tibet, China | Anti-inflammation | (Yin et al., 2016) |
| **327**, **328** | *Isatis indigotica* (da qing ye) | Hebei province, China | Anti-inflammation | (Li et al., 2016c) |
| **329**, **330** | *Isatis indigotica* | Unknown source | Antiviral activity | (Liu et al., 2015g) |
| **331**, **332** | *Curcuma phaeocaulis* | Chengdou, Sichuan province, China | Anti-inflammation | (Ma et al., 2015b) |
| **333**–**339** | *Hypericum monogynum* | China Pharmaceutical University, Jiangsu province, China | Anti-inflammation | (Xu et al., 2015c) |
| **340** | *Tricalysia fruticosa* | Xishuangbanna, Yunnan province, China | Anti-inflammation | (Shen et al., 2015) |
| **341** | *Murraya tetramera* | Wuming County, Guangxi Zhuang Autonomous Region, China | Anti-inflammation | (Lv et al., 2015) |
| **342**–**346** | *Dysoxylum gotadhora* | Mangrove National Forest Park, Hainan province, China | Anti-inflammation | (Jiang et al., 2015a) |
| **347**, **348** | *Aphanamixis grandifolia* Bl. | Yunnan province, China | Anti-inflammation | (Zhang et al., 2015e) |
| **349**, **350** | *Jatropha integerrima* | East campus of Sun Yat-sen University, Guangdong province, China | Anti-inflammation | (Zhu et al., 2015b) |
| **351**–**364** | *Physalis angulata* L*.* | Guangxi Zhuang Autonomous Region, China | Anti-inflammation | (Sun et al., 2016a) |
| **365**, **366** | *Fritillaria pallidiflora* | Xinjiang Uygur Autonomous Region, China | Anti-inflammation | (Li et al., 2016j) |
| **367**–**370** | *Tupistra chinensis* | Shennongjia Forest District, Hubei province, China | Anti-inflammation | (Xiang et al., 2016a) |
| **371**–**373** | *Tupistra chinensis* | Shennongjia Forest District, Hebei province, China | Anti-inflammation | (Xiang et al., 2016b) |
| **374**, **375** | *Entandrophragma angolense* | Brong Ahafo Region, Ghana | Anti-inflammation | (Zhang et al., 2016h) |
| **376**–**378** | *Angelica dahurica* cv. (Hangbaizhi) | Zhejiang province, China | Anti-inflammation | (Wei et al., 2016b) |
| **379**, **380** | *Angelica dahurica* | Anhui province, China | Anti-inflammation | (Yang et al., 2017) |
| **381**–**384** | *Cryptocarya concinna* | Guangdong province, China | Anti-inflammation | (Yang et al., 2016a) |
| **385** | *Dendrobium crepidatum* | Yunnan province China | Anti-inflammation | (Hu et al., 2016c) |
| **386** | *Litsea cubeba* | Herbal Medicinal Materials Company, Anhui province, China | Anti-inflammation | (Lin et al., 2016a) |
| **387**, **388** | *Vitex negundo* var. *heterophylla* | Huludao, Liaoning province, China | Anti-inflammation | (Hu et al., 2016b) |
| **389** | *Nauclea officinalis* | Hainan province, China | Anti-inflammation | (Chen et al., 2016a) |
| **390**–**394** | *Toona sinensis* (A. Juss.) Roem | Sichuan province, China | Anti-inflammation | (Li et al., 2016e) |
| **395**–**397** | *Quercus serrata* var. *brevipetiolata* | Dabie Mountains, Anhui province, China | Anti-inflammation | (Huang et al., 2016) |
| **398**, **399** | *Physalis minima* | Anhui province, China | Anti-inflammation | (Lin et al., 2016c) |
| **400** | *Lawsonia inermis* | Taiwan province, China | Anti-inflammation | (Yang et al., 2016b) |
| **401**, **402** | *Cassia fistula* | Unknown source | Antiviral activity | (Zhou et al., 2015c) |
| **403**–**405** | *Myrioneuron faberi* | Sichuan province, China | Antiviral activity | (Cao et al., 2015b) |
| **406**–**412** | *Munronia henryi* | Wenshan, Yunnan province, China | Antiviral activity  Cytotoxicity | (Yan et al., 2015b) |
| **413**–**415** | *Stellera chamaejasme* | Baotou, Inner Mongolia, China | Antiviral activity | (Yan et al., 2015a) |
| **416**, **417** | *Hypericum japonicum* | Unknown source | Antiviral activity | (Hu et al., 2016a) |
| **418**–**423** | *Sophora flavescens* | Shaanxi province, China | Antiviral activity | (Zhang et al., 2016l) |
| **424**–**429** | *Illicium oligandrum* | Guangxi Zhuang Autonomous Region, China | Antiviral activity | (Ma et al., 2016c) |
| **430**, **431** | *Spiraea japonica* var. *acuminata* | Yunnan province, China | Antiviral activity | (Ma et al., 2016f) |
| **432**–**434** | *Lavandula angustifolia* | Yunnan province, China | Antiviral activity | (Tang et al., 2016a) |
| **435** | *Nicotiana tabacum* | Yunnan province, China | Antiviral activity | (Shang et al., 2016) |
| **436**, **437** | *Nicotiana tabacum* | Yunnan province, China | Antiviral activity | (Shen et al., 2016) |
| **438** | *Ananas comosus* | Hainan province, china | Antibacterial activity | (Huang et al., 2015) |
| **439**, **440** | *Callistemon rigidus* | Guangdong province, China | Antibacterial activity | (Cao et al., 2016b) |
| **441**–**443** | *Ricinodendron heudelotii* | Hainan Island, China | 11β-HSD1 inhibitory activity | (Yu et al., 2015) |
| **444**–**453** | *Tadehagi triquetrum* | Hainan province, China | Increase glucose uptake | (Zhang et al., 2016i) |
| **454** | *Impatiens balsamina* Linn | Nanjing Zelang Phar. CO. Ltd., Jiangsu province, China | *α*-glucosidase inhibitory activity | (Li et al., 2015e) |
| **455** | *Morus alba* var. *tatarica* | Xinjiang province, China | *α*-glucosidase inhibitory activity | (Zhang et al., 2015m) |
| **456** | *Viburnum hupehense* | Caojian, Yunnan province, China | 11β-HSD1 inhibitory activity | (Chen et al., 2015b) |
| **457** | *Spermacoce latifolia* | South China Botanical Garden, Guangdong province, China | *α*-glucosidase inhibitory activity | (Luo et al., 2015c) |
| **458**, **459** | *Vaccaria hispanica* | Anguo, Hebei province, China | *α*-glucosidase inhibitory activity | (Zheng et al., 2015a) |
| **460**–**463** | *Phlomis tuberose* | Shangdu Town, Inner Mongolia Autonomous Region, China | *α*-glucosidase inhibitory activity | (Yang et al., 2015d) |
| **464** | *Dysoxylum mollissimum* | Ledong County, Hainan province, China | 11β-HSD1 inhibitory activity | (Zhou et al., 2015a) |
| **465**–**467** | Fruits of strawberry | Guangdong province, China | Antioxidant activity | (Yang et al., 2016d) |
| **468** | *Chukrasia tabularis* A | Hainan province, China | Enzyme beta glucuronidase inhibitory activity | (Peng et al., 2016a) |
| **469** | *Flemingia philippinensis* | Unknown source | PTP1B inhibitory activity | (Wang et al., 2016g) |
| **470**–**473** | *Eucalyptus robusta* | Guangxi Zhuang Autonomous Region, China | PTP1B inhibitory activity | (Yu et al., 2016c) |
| **474**–**476** | *Chloranthus oldhamii* | Jinggang Mountains, Jiangxi province, China | PTP1B inhibitory activity | (Xiong et al., 2015a) |
| **477**–**479** | *Artocarpus nanchuanensis* S.S.Chang | Jinfoshan Mountain, Chongqing, China | PTP1B inhibitory activity | (Zhang et al., 2015j) |
| **480** | *Portulaca oleracea* L. | Shandong province, China | Antioxidant activity | (Yue et al., 2015) |
| **481**, **482** | *Portulaca oleracea* | Shandong province, China | Antioxidant activity | (Jiao et al., 2015b) |
| **483**–**486** | *Portulaca oleracea* L. | Unknown source | / | (Li et al., 2016b) |
| **487**–**489** | *Murraya koenigii* (L.) Spreng | Xishuangbanna, Yunnan province, China | Antioxidant activity | (Ma et al., 2016b) |
| **490**–**496** | *Pithecellobium Clypearia* | Guangxi Zhuang Autonomous Region, China | Antioxidant activity | (Lou et al., 2016) |
| **497**–**502** | *Averrhoa carambola* L. | Guangdong province, China | Antioxidant activity | (Yang et al., 2016c) |
| **503**–**505** | *Bupleurum chinense D.C* | Unknown source | Antioxidant activity | (Li et al., 2016d) |
| **506** | *Salvia miltiorrhiza* Bge | Unknown source | Antioxidant activity | (Si et al., 2016) |
| **507** | *Salvia miltiorrhiza* | Shandong province, China | Antioxidant activity | (Li et al., 2016g) |
| **508**–**512** | *Hawthorn* | Unknown source | Antioxidant activity | (Peng et al., 2016c) |
| **513**, **514** | *Melicope ptelefolia* | Southeast Asia | Cell protecting activity | (Xu et al., 2014) |
| **515**, **516** | *Jatropha curcas* | Unknown source | Activity against TrxR | (Bao et al., 2015) |
| **517**–**522** | *Phyllanthus hainanensis* | Hainan Island, China | T and B Lymphocytes inhibitory activity | (Fan et al., 2015b) |
| **523**–**525** | *Harrisonia perforata* (Blanco) Merr. (Simaroubaceae) | Unknown source | nAChR inhibitory activity | (Fang et al., 2015) |
| **526**, **527** | *Artocarpus heterophyllus* | Nanning, Guangxi Zhuang Autonomous Region, China | Respiratory burst inhibitory activity | (Ren et al., 2015a) |
| **528** | *Salvia officinalis* | Unknown source | Cytoprotective activity | (Gong et al., 2015) |
| **529**, **530** | *Caesalpinia sappan* | Nanning, Guangxi Zhuang Autonomous Region, China | Antimalarial activity | (Ma et al., 2015a) |
| **531** | *Leonurus japonicus* | Unknown source | Vasorelaxant activity | (Xiong et al., 2015b) |
| **532**–**537** | *Toxicodendron vernicifluum* Stokes | Market of Materia Medicain Anhui province, China | Platelet aggregation inhibitory activity | (Xie et al., 2016a) |
| **538** | *Khaya senegalensis* | Guangdong province, China | Cytoprotective activity | (Tian et al., 2016c) |
| **539** | *Alangium chinense* | Guangxi Zhuang Autonomous Region, China | microsomal lipid peroxidation inhibitory activity | (Zhang et al., 2016k) |
| **540**–**543** | *Lespedeza cuneata* | Henan province, China | Active towards the transcription of xbp1 | (Zhou et al., 2016b) |
| **544**–**553** | *Hypericum uralum* | Yunnan province, China | Cytoprotective activity | (Zhou et al., 2016e) |
| **554**, **555** | *Sassafras tzumu* | Guangxi Zhuang Autonomous Region, China | AChE inhibitoryory activity | (Lu et al., 2017) |
| **556** | *Schisandra pubescens* | Jinfo mountain, Chongqing, China | Hepatoprotective activitiy | (Wang et al., 2016a) |
| **557** | *Celastrus monospermus* | Guangdong province, China | Lifespan extending activity | (Gao et al., 2016a) |
| **558**, **559** | *Pteris cretica* L. | Guizhou province, China | Lipid-lowering activity | (Luo et al., 2016b) |
| **560**–**563** | *Cimicifuga yunnanensis* | Daocheng County, Sichuan province, China | Anti-angiogenic activity | (Nian et al., 2015) |
| **564**, **565** | *Callicarpa macrophylla* | Guangxi Zhuang Autonomous Region, China | / | (Xu et al., 2015a) |
| **566**–**571** | *Xanthium sibiricum* | Helen City, Heilongjiang province, China | / | (Shi et al., 2015) |
| **572**–**577** | *Callistemon viminalis* | Guangdong province, China | / | (Wu et al., 2015f) |
| **578** | *Ainsliaea fragrans* | Shiyan City, Hubei province, China | / | (Xue et al., 2015) |
| **579**, **580** | *Macaranga denticulate* | Unknown source | / | (Yang et al., 2015b) |
| **581**, **582** | *Carpesium abrotanoides* L. | Unknown source | / | (Wu et al., 2015b) |
| **583**–**585** | *Isodon rubescens* | Jianshi County, Hubei province, China | / | (Liu et al., 2015f) |
| **586**–**590** | *Ervatamia hainanensis* | Tunchang County, Hainan province, China | / | (Zhang et al., 2015c) |
| **591**, **592** | *Flueggea virosa* | Unknown source | / | (Zhang et al., 2015f) |
| **593**–**596** | *Forsythia suspense* | Yuncheng City, Shanxi province, China | / | (Zhang et al., 2015d) |
| **597**–**600** | *Gelsemium elegans* | Conghua city, Guangdong province, China | / | (Zhang et al., 2015k) |
| **601**–**604** | *Gelsemium elegans* | Xishuangbanna Tropical Botanical Garden, Yunnan province, China | / | (Xu et al., 2015d) |
| **605**, **606** | *Glycosmis pentaphylla* | Unknown source | / | (Chen et al., 2015c) |
| **607**–**609** | *Haplomitrium mnioides* | Guizhou province, China | / | (Zhou et al., 2016c) |
| **610**, **611** | *Haplomitrium mnioides* | Unknown source | / | (Zhou et al., 2015b) |
| **612** | *Hypericum attenuatum* Choisy | Qichun County, Hubei province, China | / | (Li et al., 2015a) |
| **613** | *Icacina trichantha* | Unknown source | / | (Zhao et al., 2015a) |
| **614**, **615** | *Stahlianthus involucratus* | Unknown source | / | (Li et al., 2015d) |
| **616**, **617** | *Kadsura coccinea* | Ziyuan, Guangxi Zhuang Autonomous Region, China | / | (Liang et al., 2015) |
| **618** | *Kadsura coccinea* | Unknown source | / | (Hu et al., 2016d) |
| **619**–**621** | *Kadsura coccinea* | Unknown source | / | (Hu et al., 2015) |
| **622**–**629** | *Daphne genkwa* | Mianyang, Sichuan province, China | / | (Li et al., 2015c) |
| **630**, **631** | *Cinnamomum subavenium* | Laifeng, Hubei province, China | / | (Lai et al., 2015) |
| **632**–**634** | *Scrophularia ningpoensis* | Unknown source | / | (Zhang et al., 2015g) |
| **635**–**638** | *Penthorum chinense* | Guling, Sichuan province, China | / | (He et al., 2015) |
| **639**–**649** | *Hypericum henryi* | Hongtudi in Dongchuan Prefecture, Yunnan province, China | / | (Yang et al., 2015c) |
| **650**–**653** | *Iris tectorum* | Unknown source | / | (Zhang et al., 2015a) |
| **654**–**657** | *Callicarpa randaiensis* | Nantou County, Taiwan province, China | / | (Cheng et al., 2015) |
| **658**, **659** | *Abies chensiensis* | Unknown source | / | (Zhao et al., 2015b) |
| **660**, **661** | *Rhododendron capitatum* | Unknown source | / | (Liao et al., 2015a) |
| **662**, **663** | *Garcinia multiflora* | Unknown source | / | (Fan et al., 2015a) |
| **664**, **665** | *Garcinia multiflora* | Unknown source | / | (Tian et al., 2016a) |
| **666**, **667** | *Isodon* *phyllostachys* | Sichuan province, China | / | (Yang J et al., 2016e) |
| **668**–**670** | *Alstonia scholaris* | Unknown source | / | (Pan et al., 2016b) |
| **671**–**673** | *Lycopodium annotinum* | Unknown source | / | (Tang et al., 2016b) |
| **674**–**676** | *Aphanamixis grandifolia* | Hainan Island, China | / | (Zhang et al., 2016b) |
| **677**, **678** | *Artabotrys hexapetalus* | Unknown source | / | (Xi et al., 2016) |
| **679**–**683** | *Salvia plebeian* | Unknown source | / | (Xu et al., 2016b) |
| **684**–**687** | *Garcinia oligantha* | Unknown source | / | (Tang et al., 2016c) |
| **688**–**691** | *Cephalotaxus sinensis* | Guangxi Zhuang Autonomous Region, China | / | (Xu et al., 2016b) |
| **692**–**694** | *Toona ciliate* | Unknown source | / | (Liu et al., 2016a) |
| **695**–**697** | *Cipadessa cinerascens* | Unknown source | / | (Yu et al., 2016b) |
| **698** | *Picrasma quassioides* | Jiangxi province, China | / | (Xu et al., 2016c) |
| **699** | *Incarvillea delavayi* | Yunnan province, China | / | (Zhang et al., 2016n) |
| **700**–**702** | *Dryopteris championii* | Hainan province, China | / | (Chen et al., 2016d) |
| **703** | *Euphorbia kansui* | Unknown source | / | (Fei et al., 2016) |
| **704** | *Psidium guajava* | Unknown source | / | (Li et al., 2016a) |
| **705**, **706** | *Daphniphyllum himalense* | Unknown source | / | (Zhang et al., 2016c) |
| **707** | *Schisandra lancifolia* | Unknown source | / | (Shi et al., 2016) |
| **708**–**710** | *Myrioneuron tonkinensis* | Guangxi Zhuang Autonomous Region, China | / | (Li et al., 2016i) |
| **711** | *Phlegmariurus fargesii* | Unknown source | / | (Meng et al., 2016b) |
| **712**, **713** | *Rhodomyrtus tomentosa* | Jiangxi province, China | / | (Liu et al., 2016b) |
| **714** | *Schisandra incarnate* | Hubei province, China | / | (Zhou et al., 2016d) |
| **715**, **716** | *Trichilia connaroides* | Unknown source | / | (An et al., 2016a) |
| **717**, **718** | *Trichilia connaroides* | Unknown source | / | (An et al., 2016c) |
| **719** | *Zephyranthes candida* | Hubei province, China | / | (Zhan et al., 2016) |
| **720** | *Cephalotaxus lanceolata* | Unknown source | / | (Ni et al., 2016) |
| **721** | *Valeriana officinalis* var. *latiofolia* | Guizhou province, China | / | (Wang et al., 2015c) |
| **722**–**729** | Toad *Bufo bufo gargarizans* | Unknown source | Cytotoxicity | (Zhang et al., 2016g) |
| **730** | *Plasmodium falciparum* | Guangxi Zhuang Autonomous Region, China | Nanomolar *P. falciparum* growth inhibitoryion activity | (Zhou et al., 2017) |
| Note: ^a^/: no (significant) bioactivity was reported. | | | | |
|  |  |  |  |  |
|  |  |  |  |  |
|  |  |  |  |  |
|  |  |  |  |  |
|  |  |  |  |  |
|  |  |  |  |  |
|  |  |  |  |  |
|  | | | | |
